# Supplementary material for: The Power of Social Media in the Promotion and Tenure of Clinician Educators
Source: MedEdPORTAL. 2020 Aug 10;16:10943. doi: 10.15766/mep_2374-8265.10943 (PMC7431188; doi:10.15766/mep_2374-8265.10943)
Supplement: Supplementary file 1 — Content Slides.pptxEvaluation Form.docx [file mep_2374-8265.10943-s001.zip › A. Content Slides.pptx]

## Slide 1
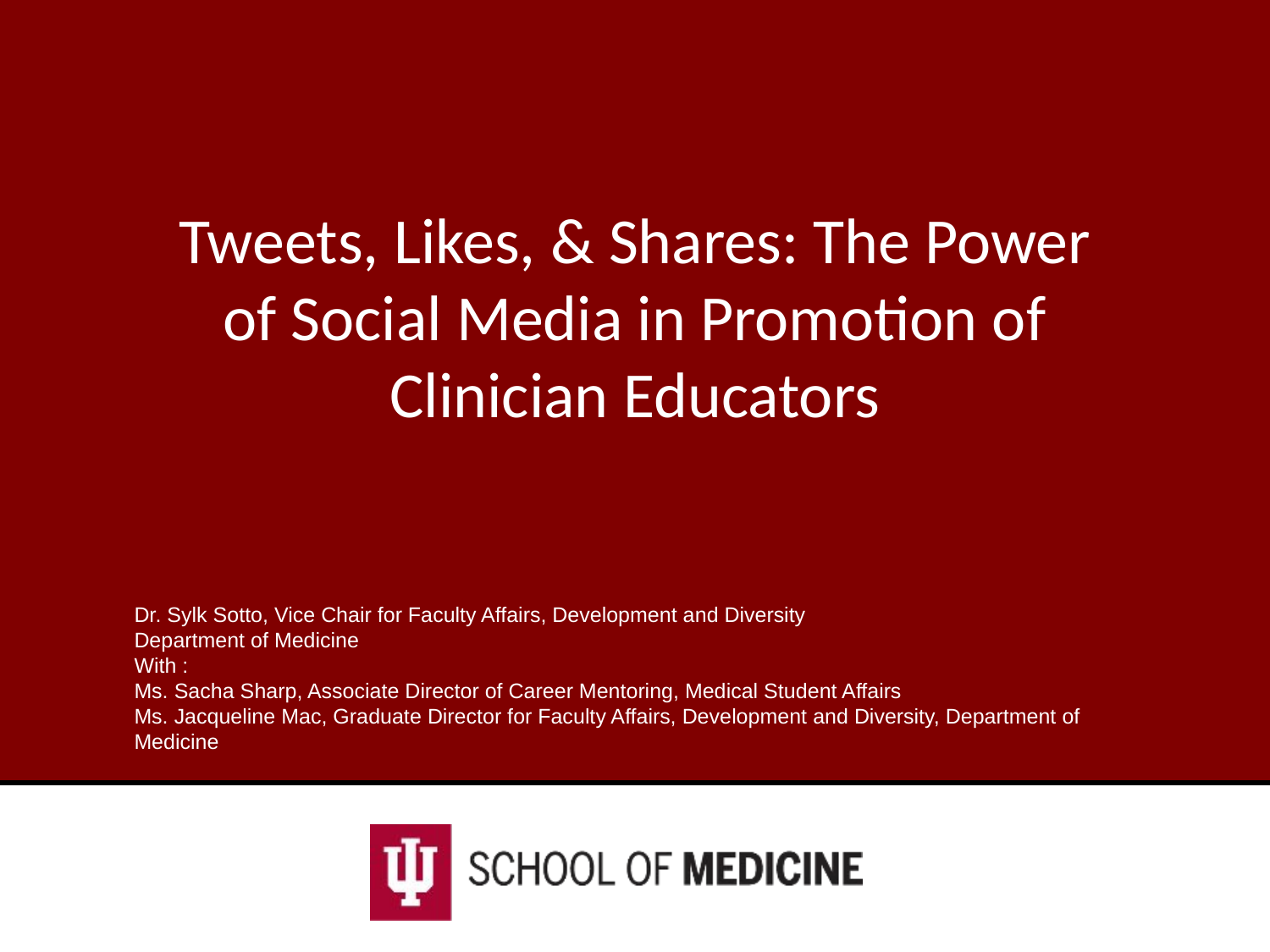

# Tweets, Likes, & Shares: The Power of Social Media in Promotion of Clinician Educators
Dr. Sylk Sotto, Vice Chair for Faculty Affairs, Development and Diversity
Department of Medicine
With :
Ms. Sacha Sharp, Associate Director of Career Mentoring, Medical Student Affairs
Ms. Jacqueline Mac, Graduate Director for Faculty Affairs, Development and Diversity, Department of Medicine

## Slide 2
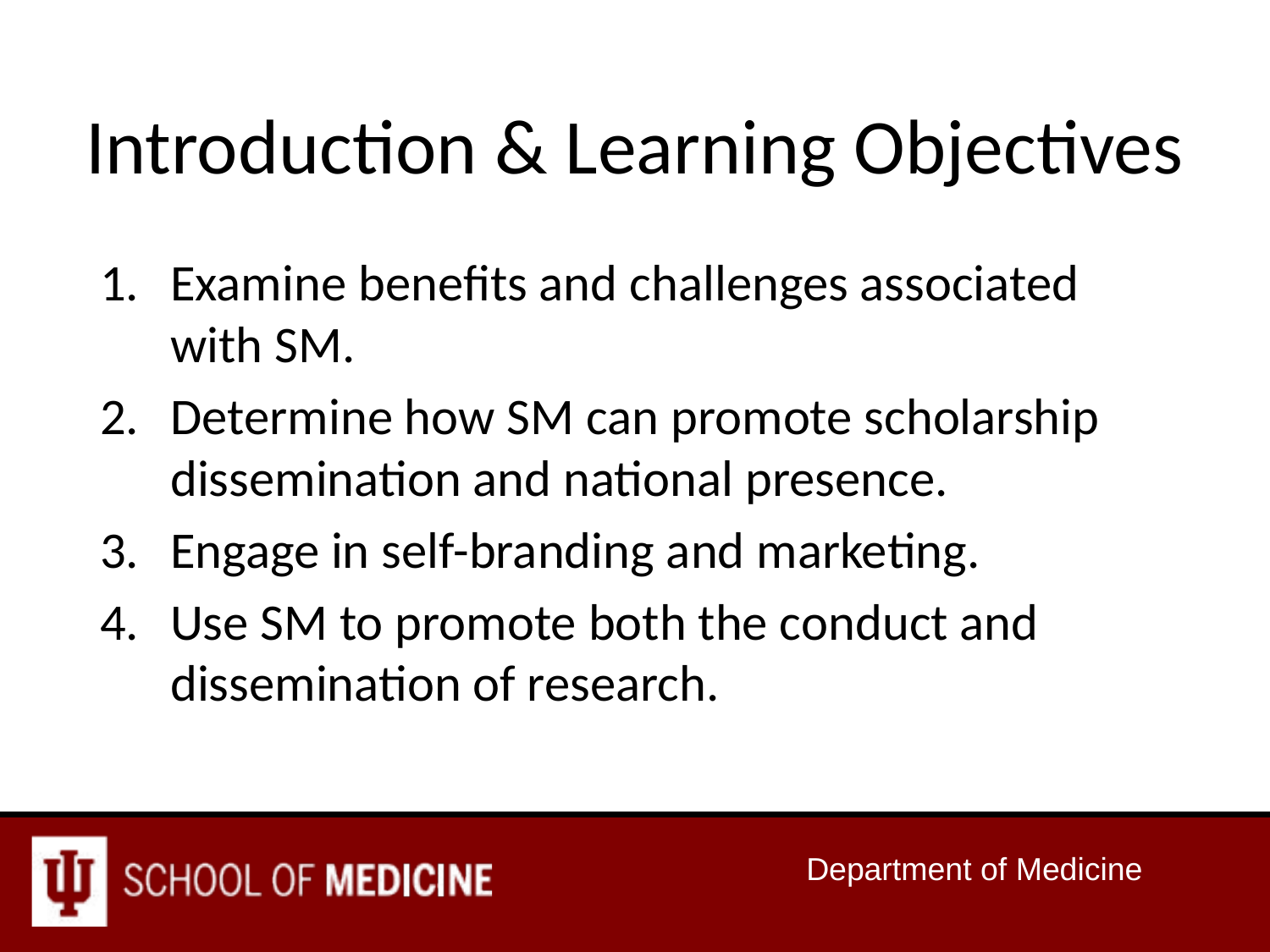

# Introduction & Learning Objectives
Examine benefits and challenges associated with SM.
Determine how SM can promote scholarship dissemination and national presence.
Engage in self-branding and marketing.
Use SM to promote both the conduct and dissemination of research.
Department of Medicine

## Slide 3
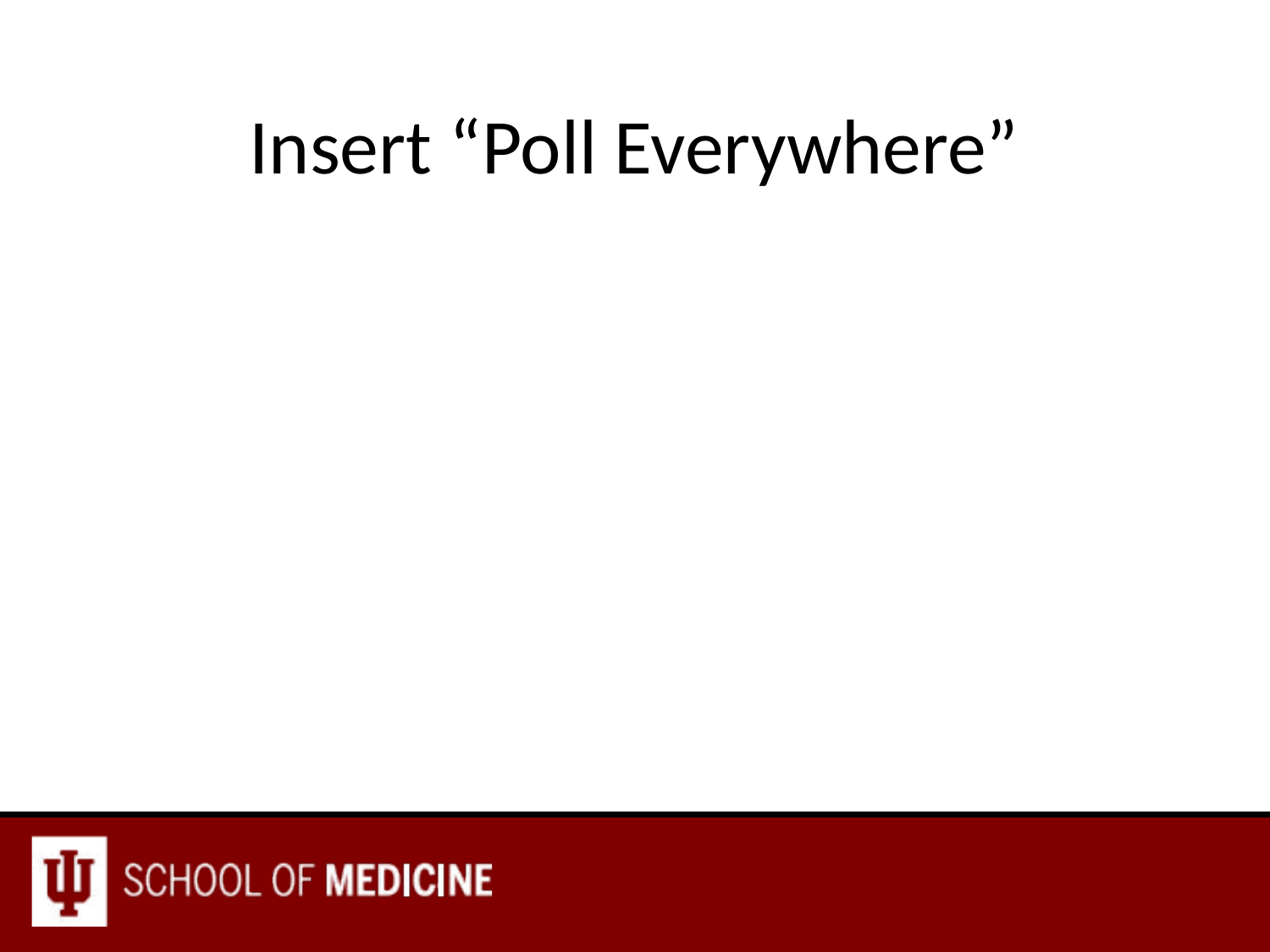

# Insert “Poll Everywhere”

## Slide 4
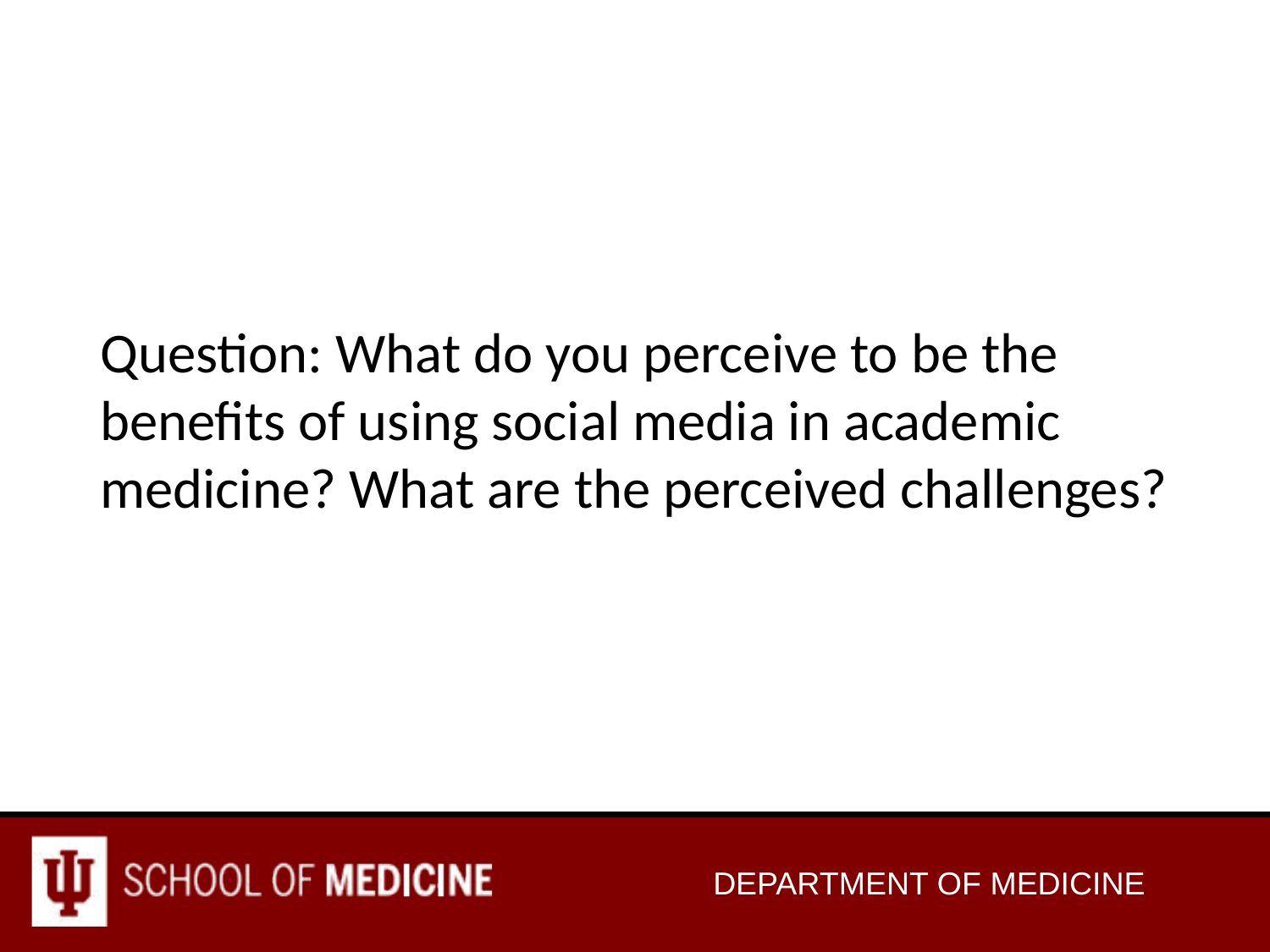

Question: What do you perceive to be the benefits of using social media in academic medicine? What are the perceived challenges?
DEPARTMENT OF MEDICINE

## Slide 5
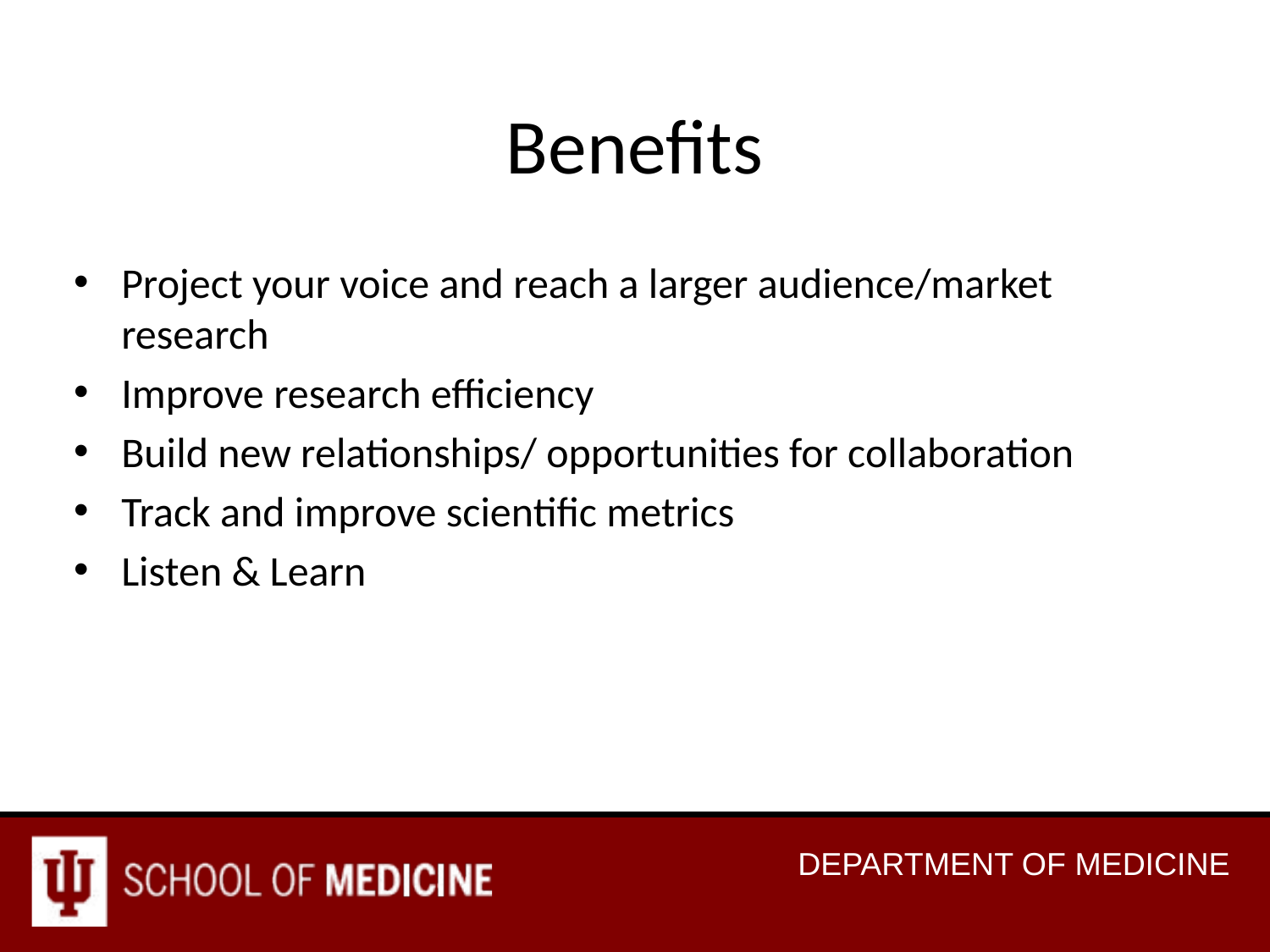

# Benefits
Project your voice and reach a larger audience/market research
Improve research efficiency
Build new relationships/ opportunities for collaboration
Track and improve scientific metrics
Listen & Learn
DEPARTMENT OF MEDICINE

## Slide 6
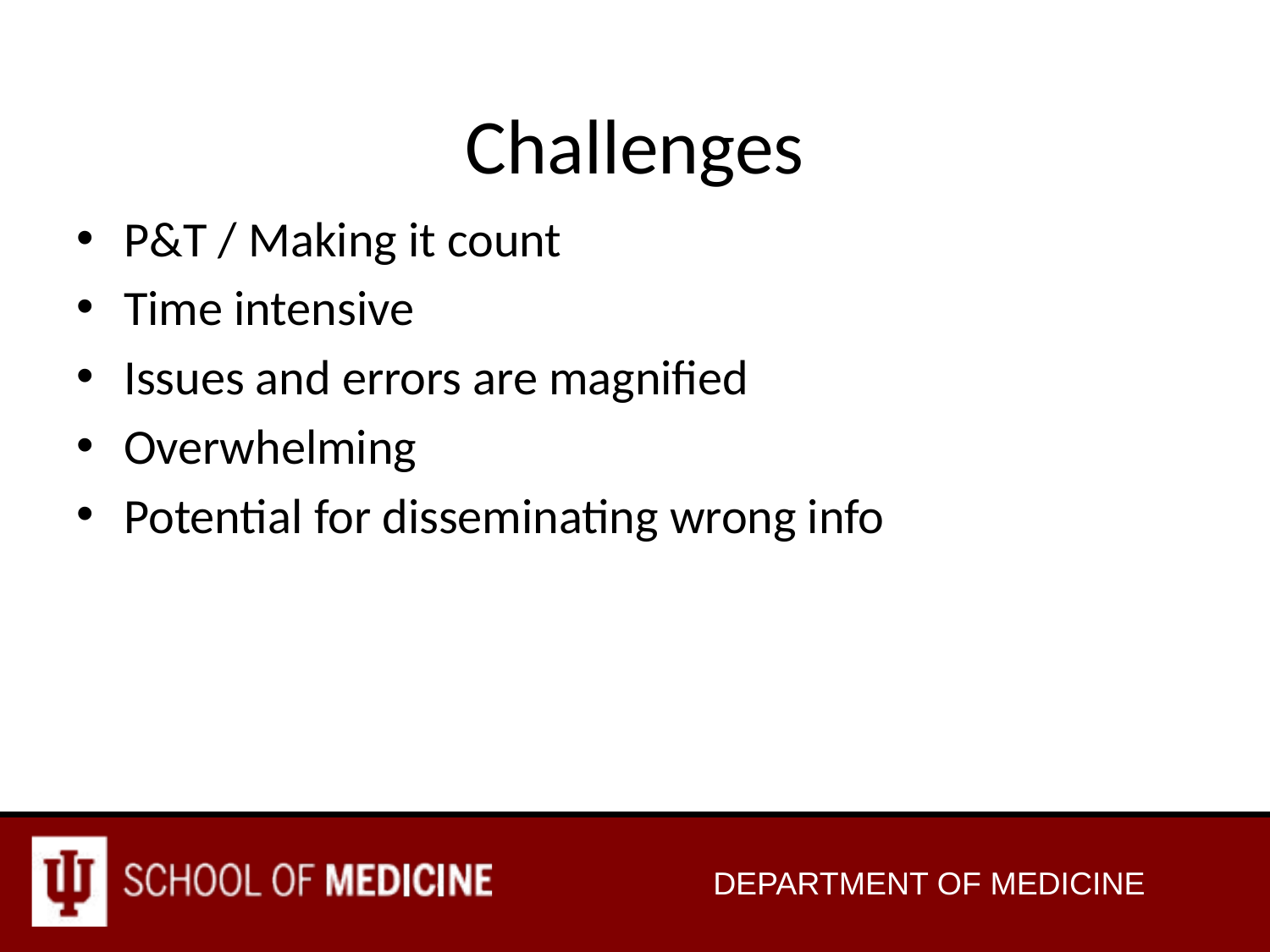

# Challenges
P&T / Making it count
Time intensive
Issues and errors are magnified
Overwhelming
Potential for disseminating wrong info
DEPARTMENT OF MEDICINE

## Slide 7
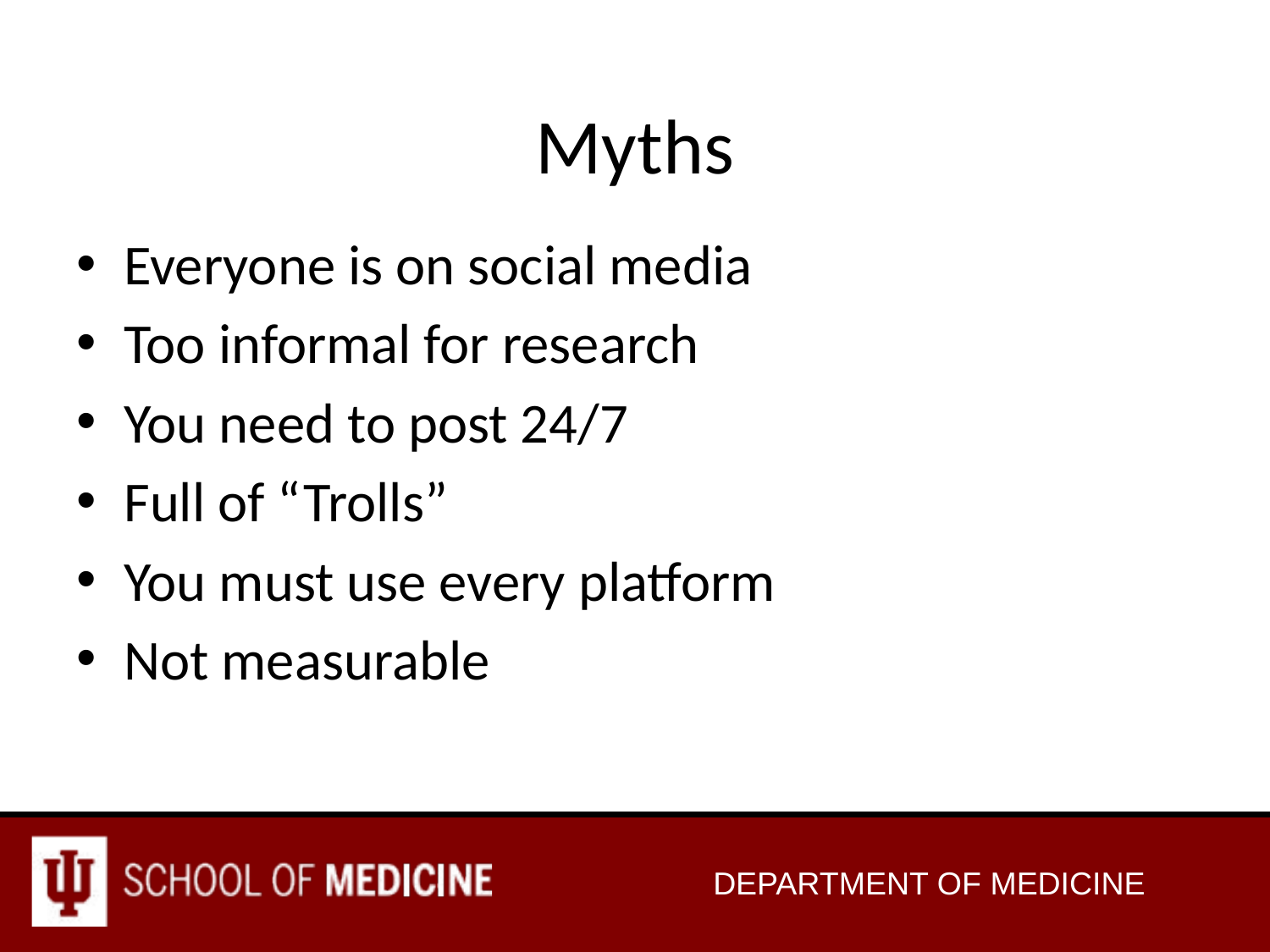

# Myths
Everyone is on social media
Too informal for research
You need to post 24/7
Full of “Trolls”
You must use every platform
Not measurable
DEPARTMENT OF MEDICINE

## Slide 8
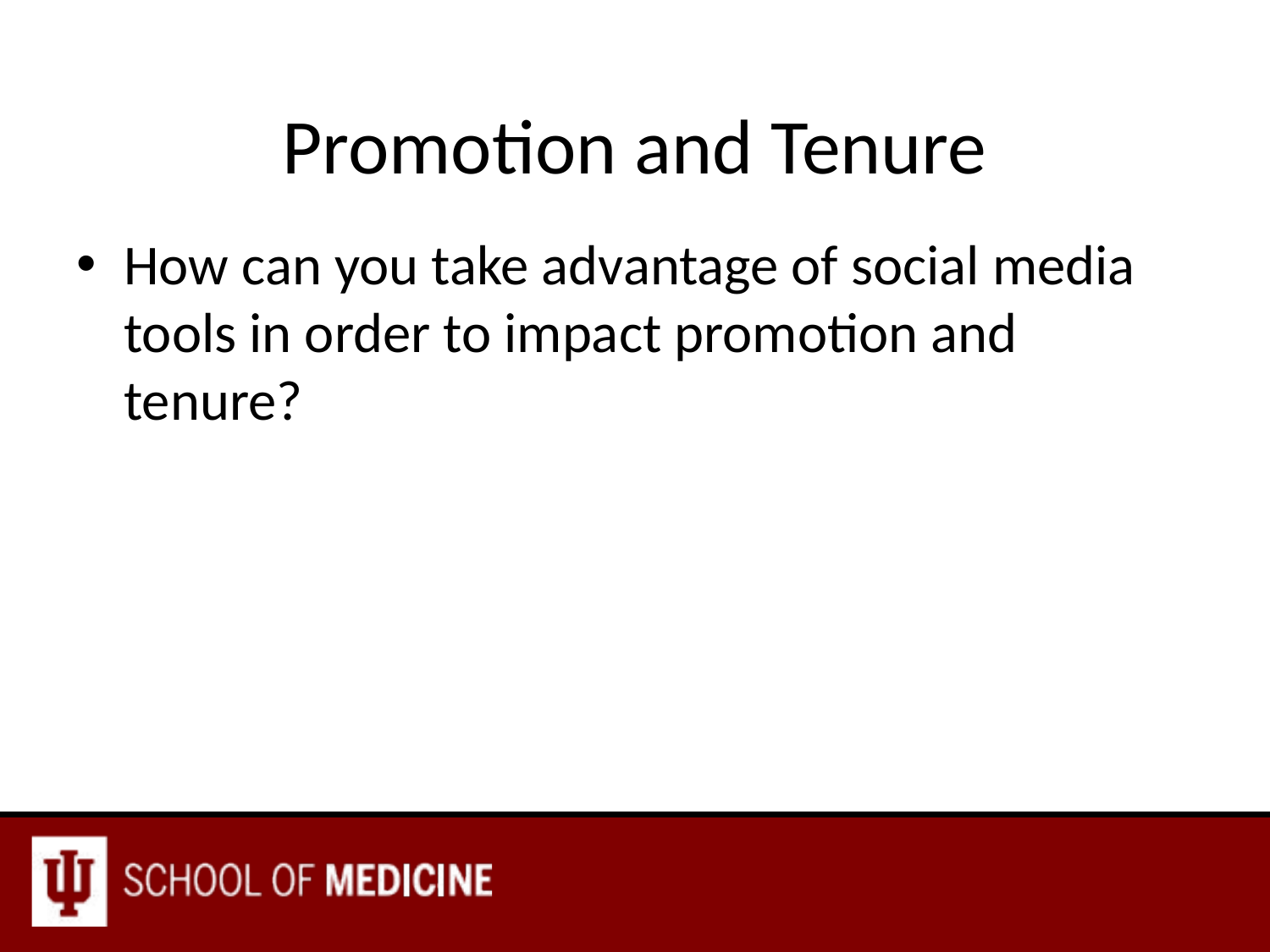

# Promotion and Tenure
How can you take advantage of social media tools in order to impact promotion and tenure?

## Slide 9
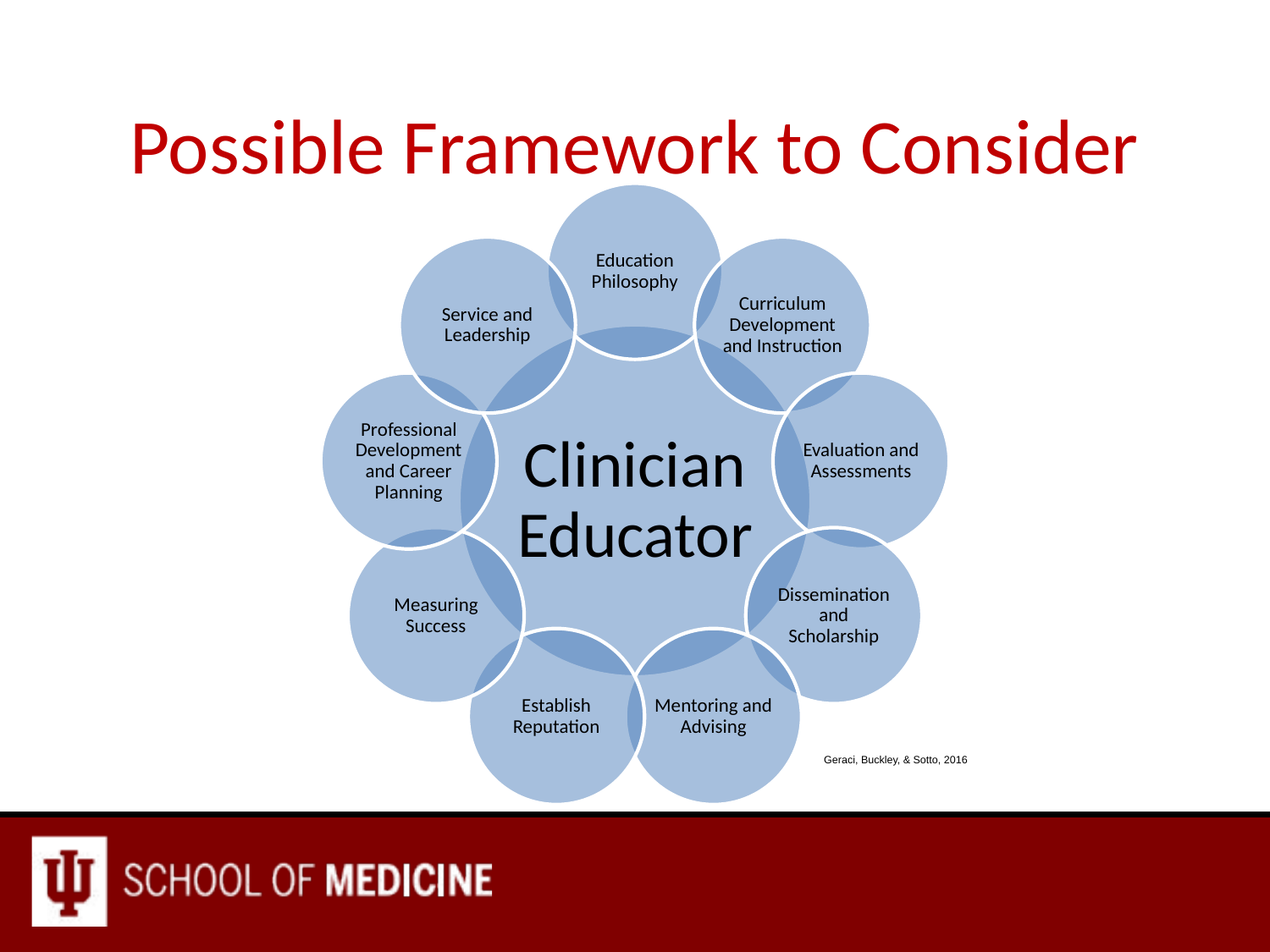

# Possible Framework to Consider
Geraci, Buckley, & Sotto, 2016

## Slide 10
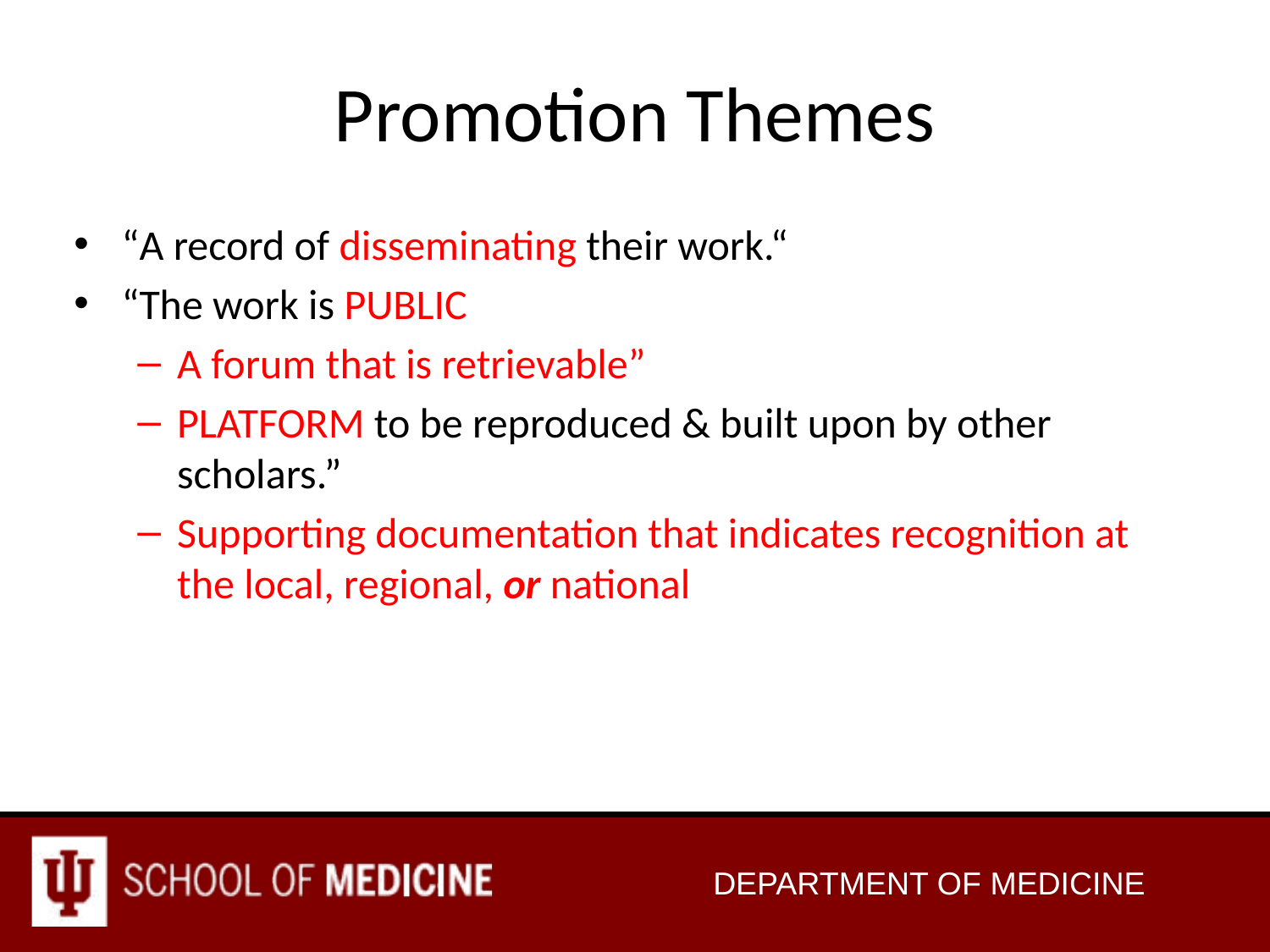

# Promotion Themes
“A record of disseminating their work.“
“The work is PUBLIC
A forum that is retrievable”
PLATFORM to be reproduced & built upon by other scholars.”
Supporting documentation that indicates recognition at the local, regional, or national
DEPARTMENT OF MEDICINE

## Slide 11
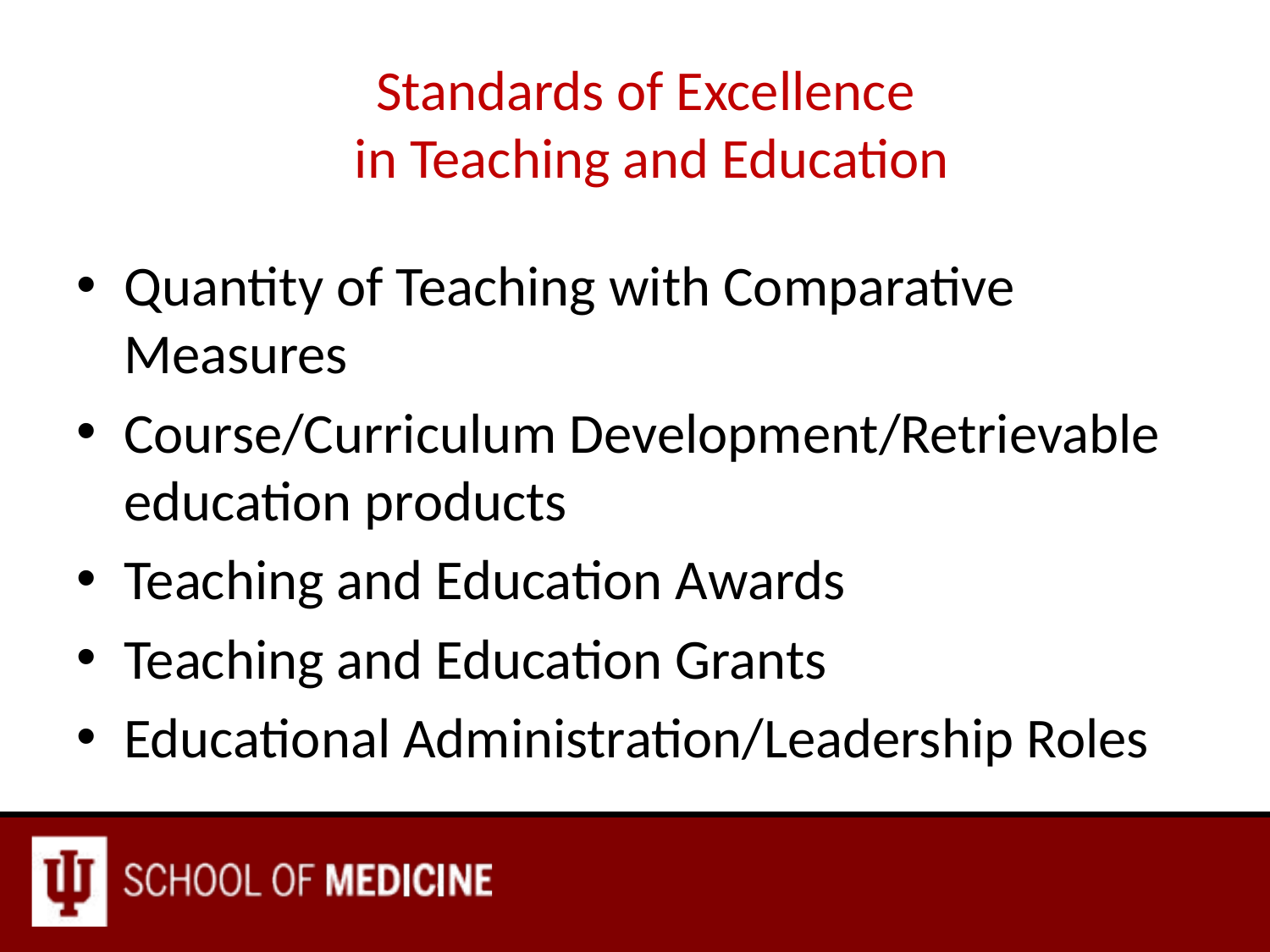

# Standards of Excellence in Teaching and Education
Quantity of Teaching with Comparative Measures
Course/Curriculum Development/Retrievable education products
Teaching and Education Awards
Teaching and Education Grants
Educational Administration/Leadership Roles

## Slide 12
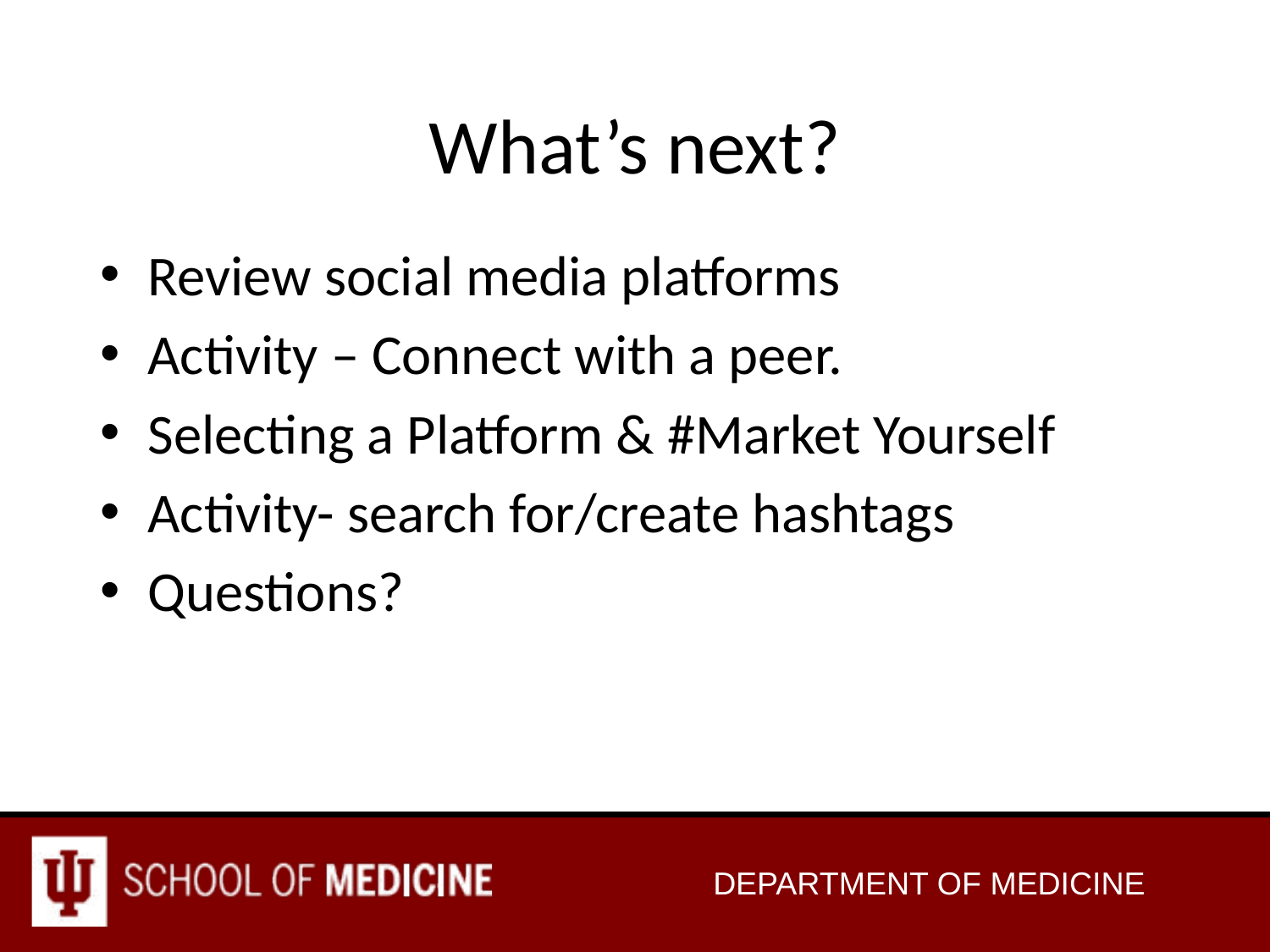

# What’s next?
Review social media platforms
Activity – Connect with a peer.
Selecting a Platform & #Market Yourself
Activity- search for/create hashtags
Questions?
DEPARTMENT OF MEDICINE

## Slide 13
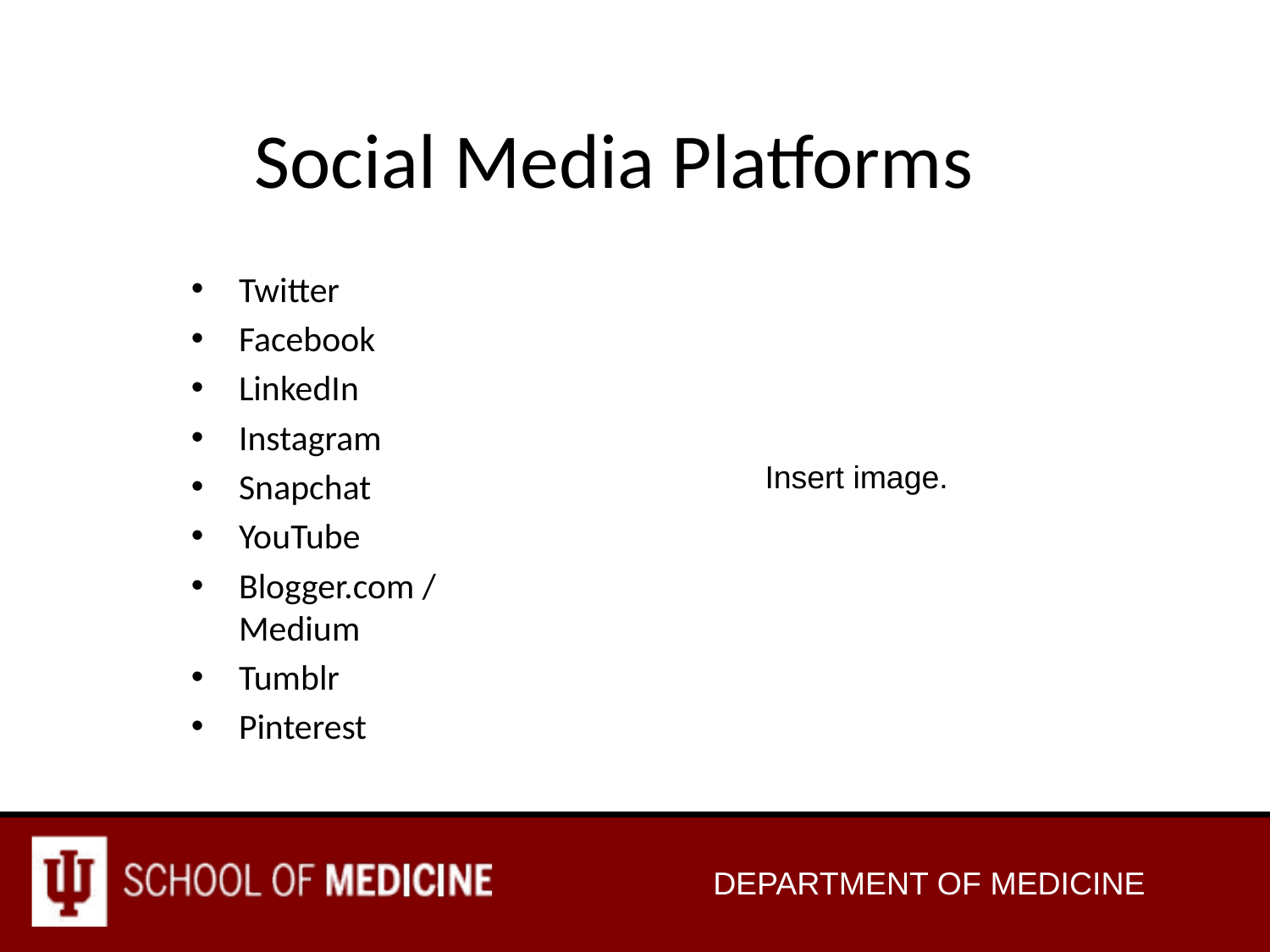

# Social Media Platforms
Twitter
Facebook
LinkedIn
Instagram
Snapchat
YouTube
Blogger.com / Medium
Tumblr
Pinterest
Insert image.
DEPARTMENT OF MEDICINE

## Slide 14
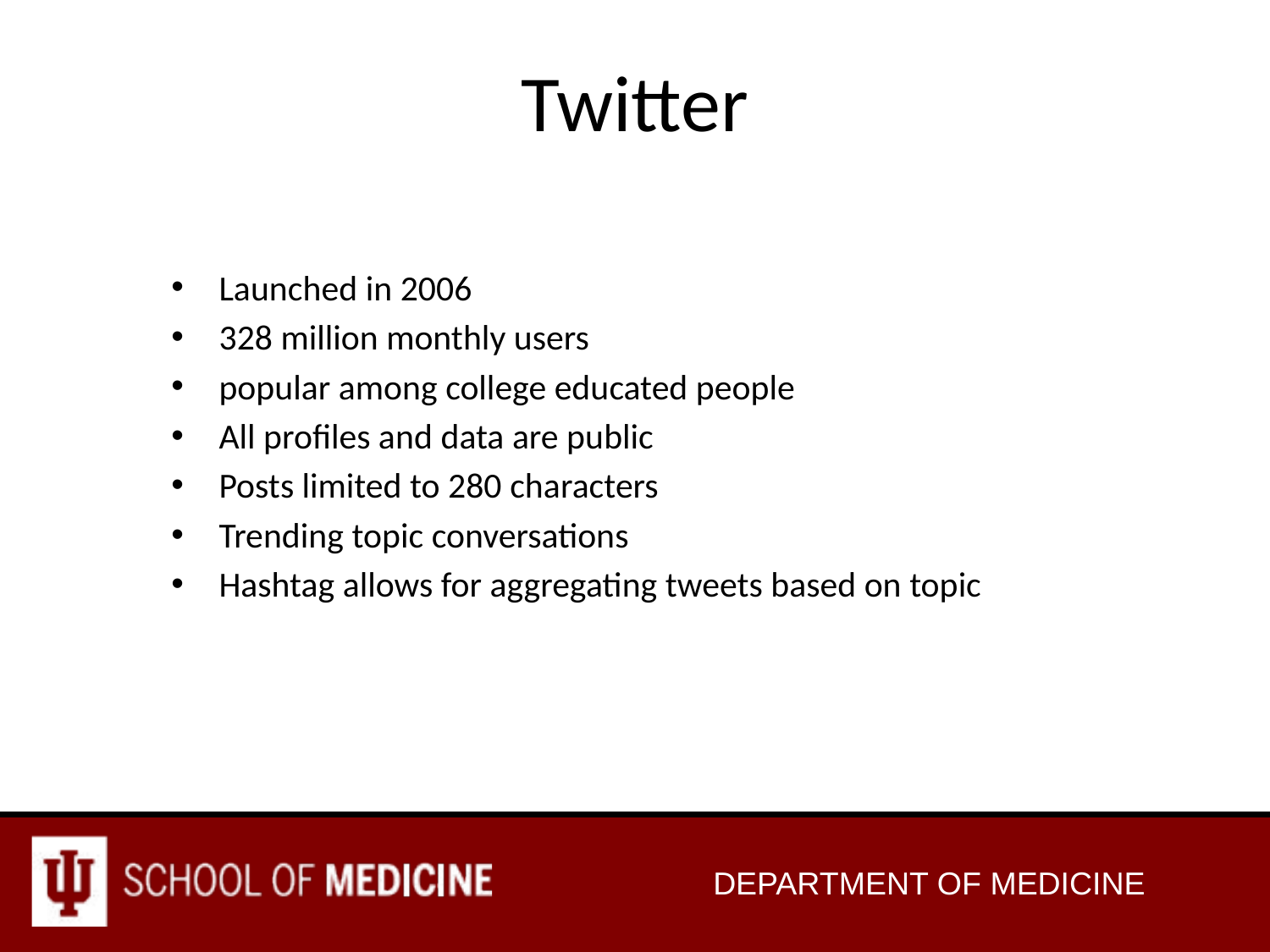

# Twitter
Launched in 2006
328 million monthly users
popular among college educated people
All profiles and data are public
Posts limited to 280 characters
Trending topic conversations
Hashtag allows for aggregating tweets based on topic
DEPARTMENT OF MEDICINE

## Slide 15
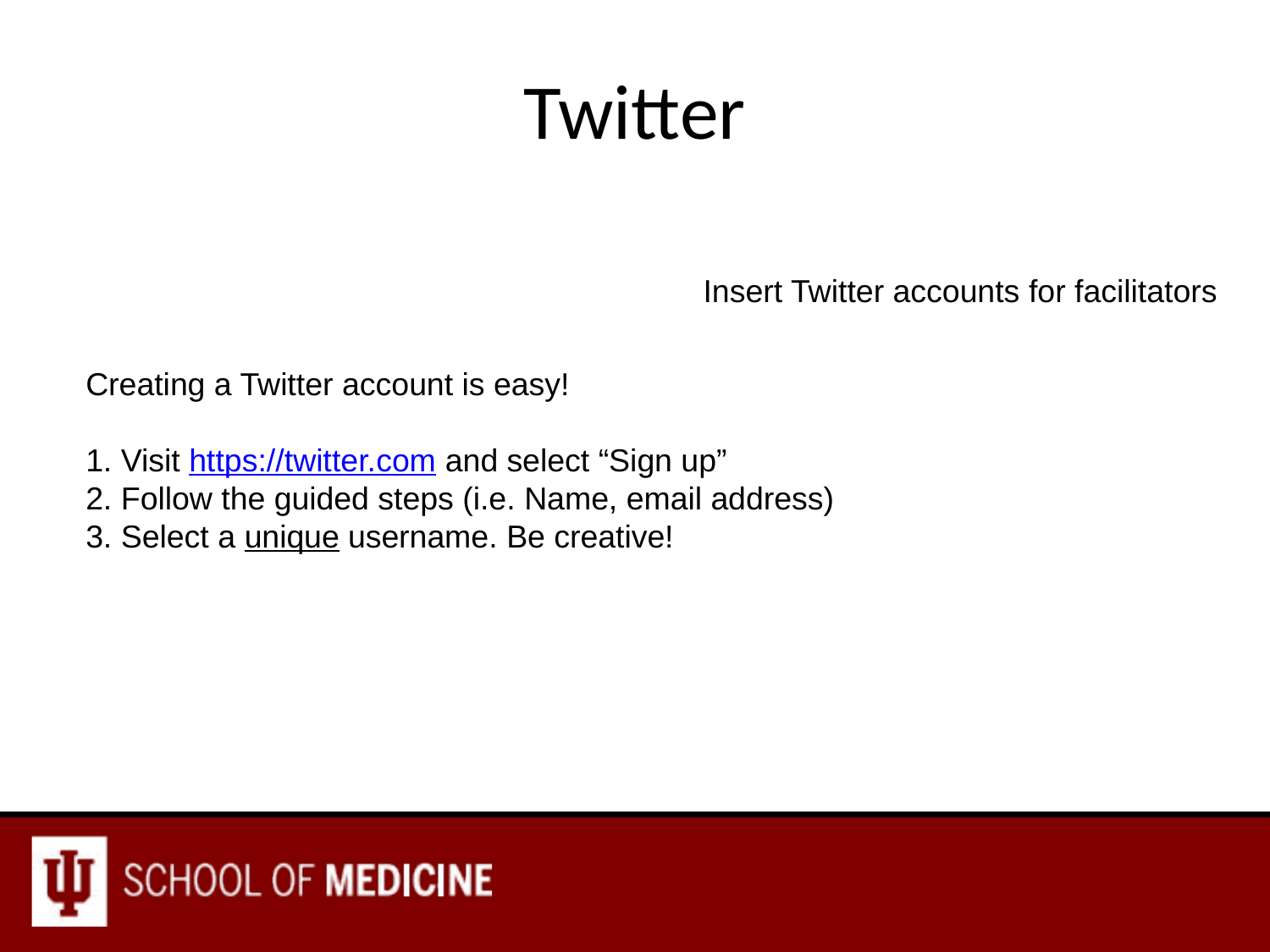

# Twitter
Insert Twitter accounts for facilitators
Creating a Twitter account is easy!
1. Visit https://twitter.com and select “Sign up”
2. Follow the guided steps (i.e. Name, email address)
3. Select a unique username. Be creative!

## Slide 16
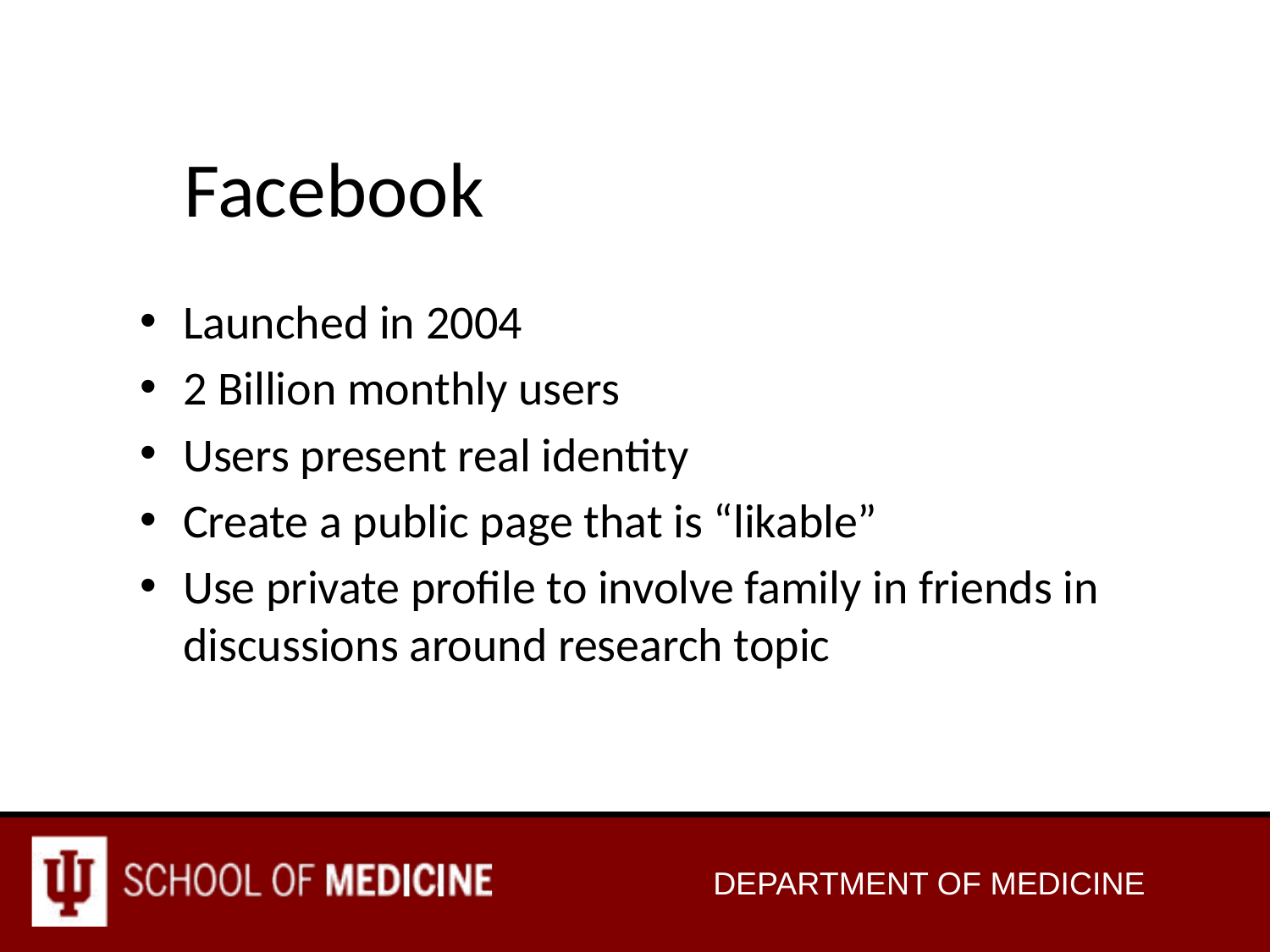

# Facebook
Launched in 2004
2 Billion monthly users
Users present real identity
Create a public page that is “likable”
Use private profile to involve family in friends in discussions around research topic
DEPARTMENT OF MEDICINE

## Slide 17
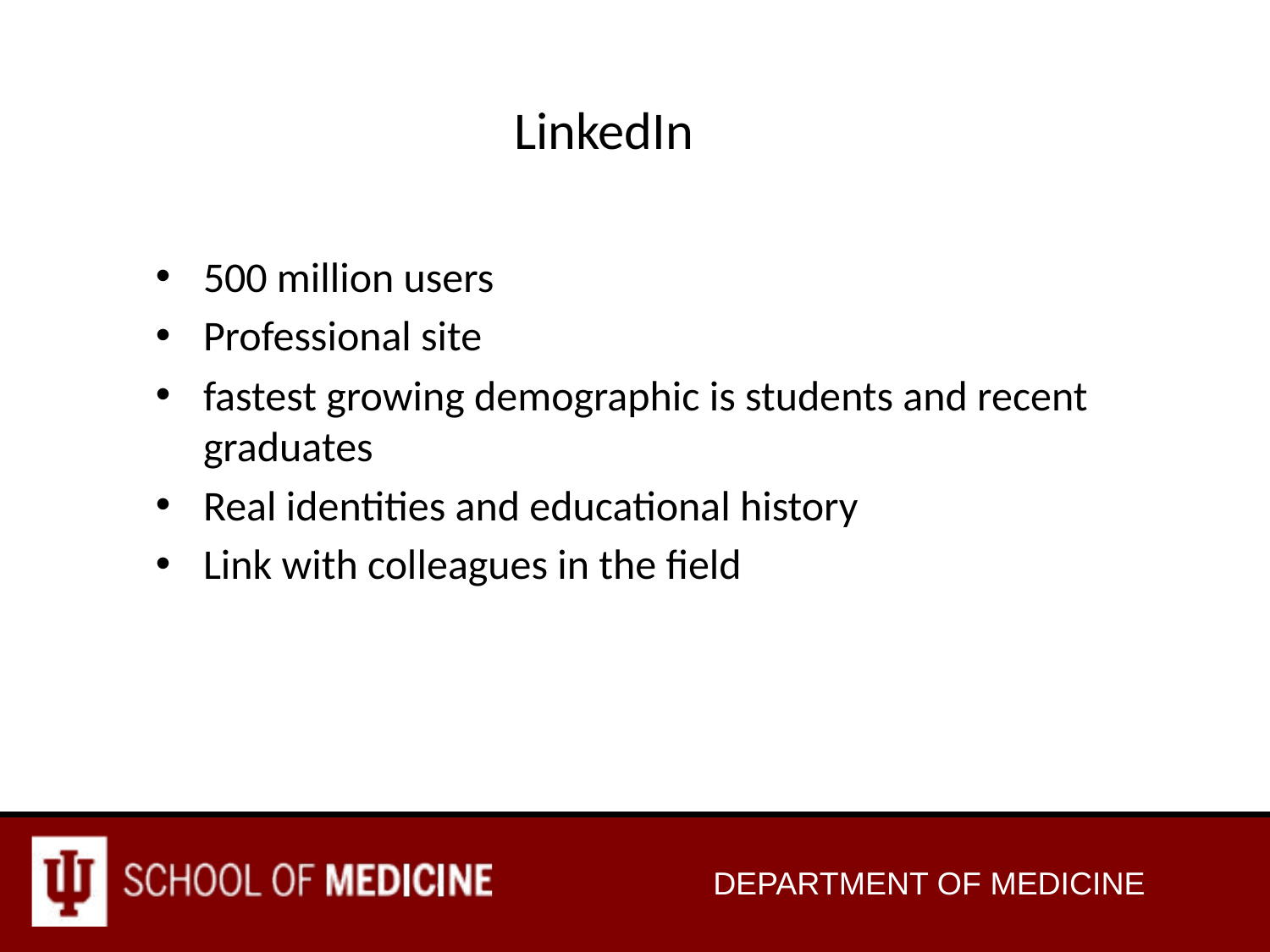

LinkedIn
500 million users
Professional site
fastest growing demographic is students and recent graduates
Real identities and educational history
Link with colleagues in the field
DEPARTMENT OF MEDICINE

## Slide 18
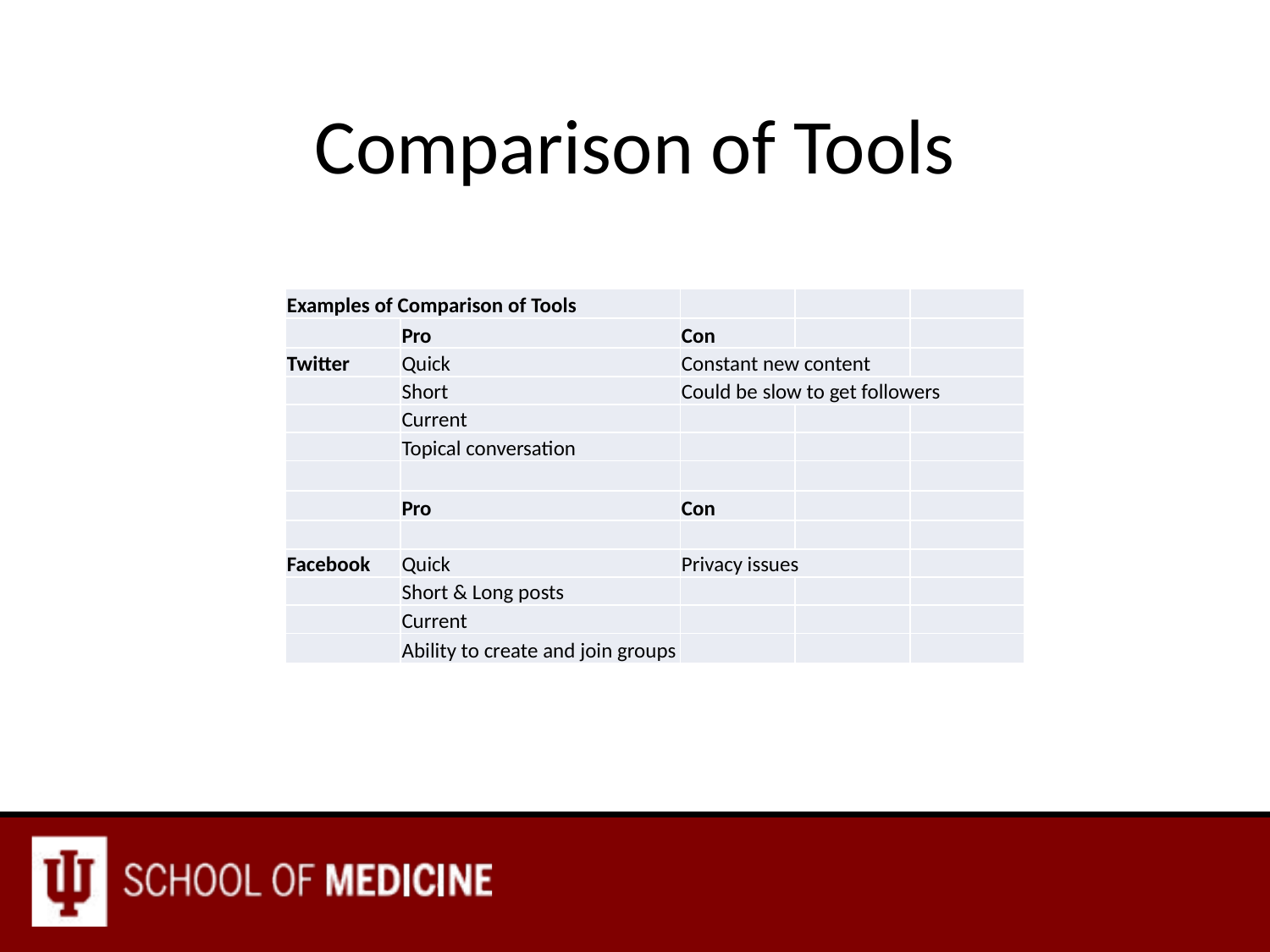

# Comparison of Tools
| Examples of Comparison of Tools | | | | |
| --- | --- | --- | --- | --- |
| | Pro | Con | | |
| Twitter | Quick | Constant new content | | |
| | Short | Could be slow to get followers | | |
| | Current | | | |
| | Topical conversation | | | |
| | | | | |
| | Pro | Con | | |
| | | | | |
| Facebook | Quick | Privacy issues | | |
| | Short & Long posts | | | |
| | Current | | | |
| | Ability to create and join groups | | | |

## Slide 19
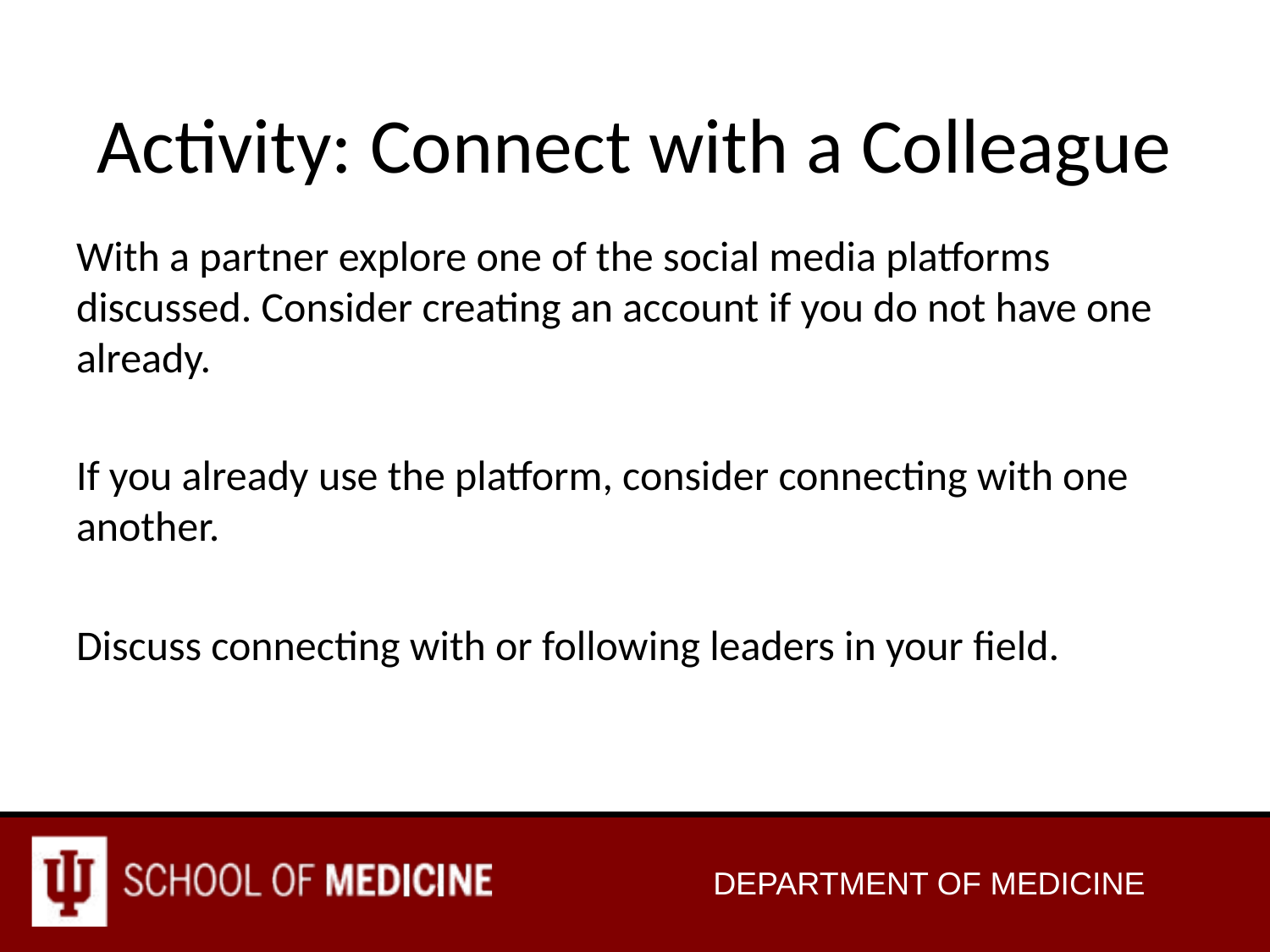

# Activity: Connect with a Colleague
With a partner explore one of the social media platforms discussed. Consider creating an account if you do not have one already.
If you already use the platform, consider connecting with one another.
Discuss connecting with or following leaders in your field.
DEPARTMENT OF MEDICINE

## Slide 20
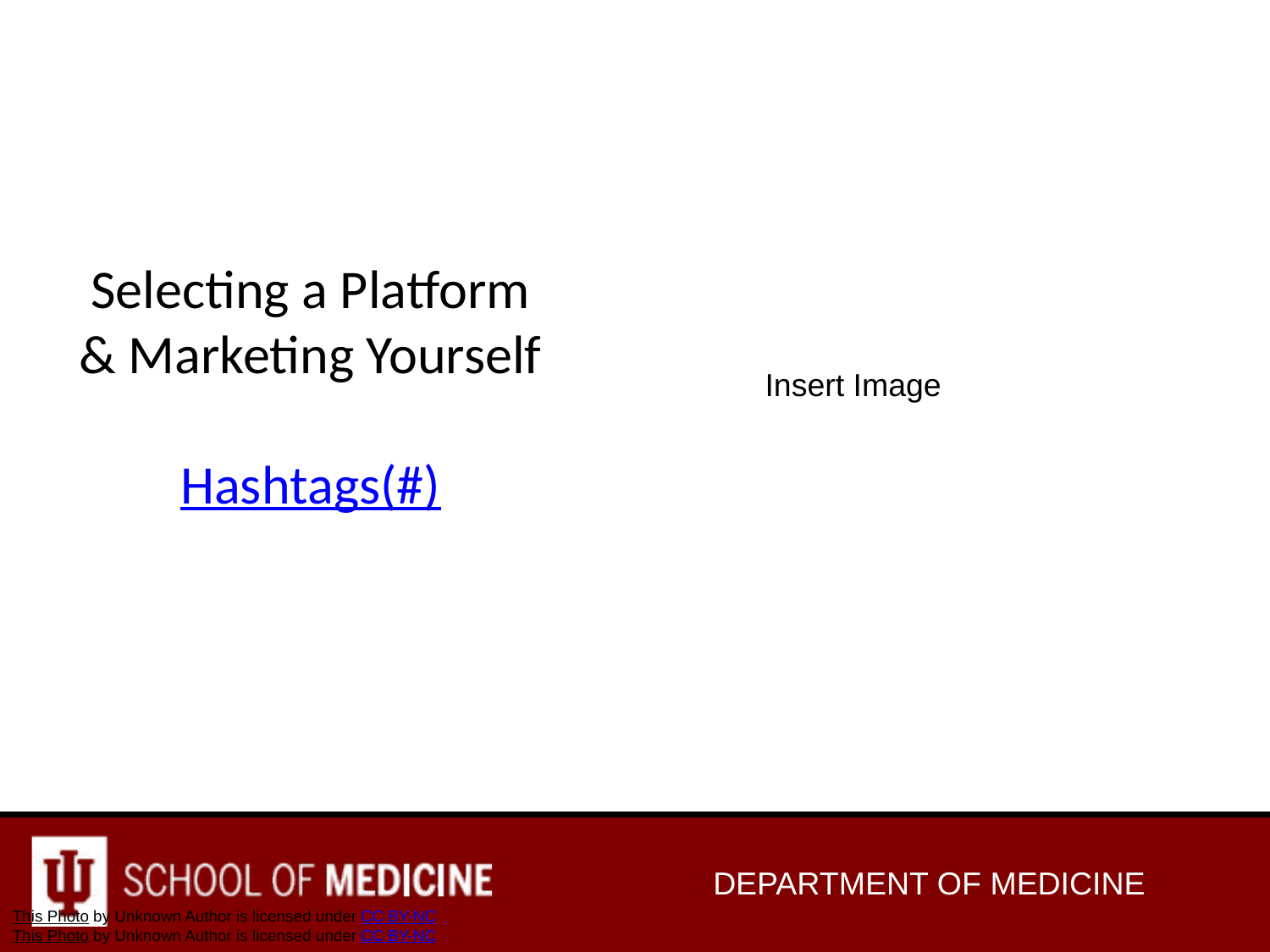

# Selecting a Platform & Marketing Yourself Hashtags(#)
Insert Image
DEPARTMENT OF MEDICINE
This Photo by Unknown Author is licensed under CC BY-NC
This Photo by Unknown Author is licensed under CC BY-NC

## Slide 21
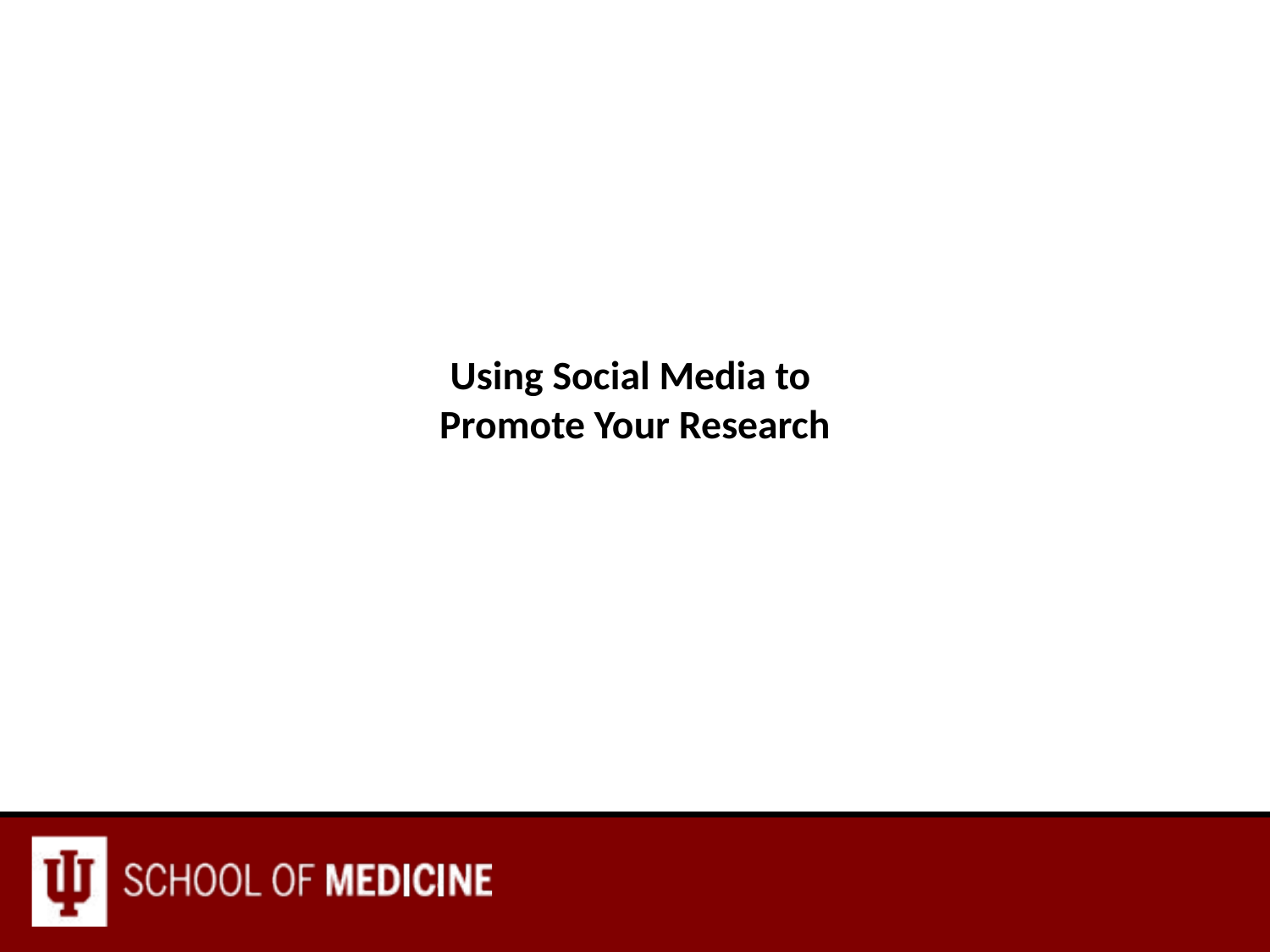

# Using Social Media to Promote Your Research

## Slide 22
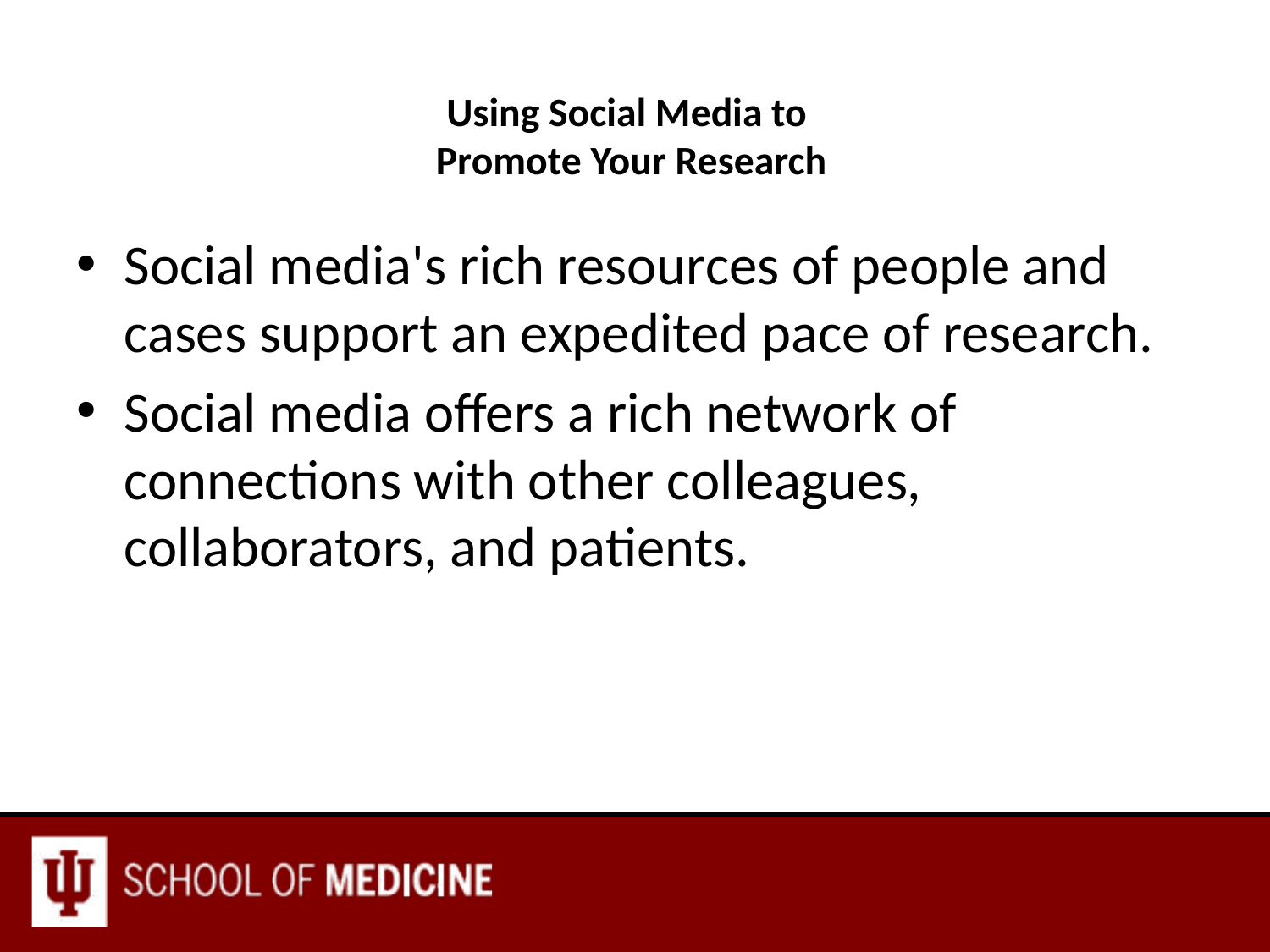

# Using Social Media to Promote Your Research
Social media's rich resources of people and cases support an expedited pace of research.
Social media offers a rich network of connections with other colleagues, collaborators, and patients.

## Slide 23
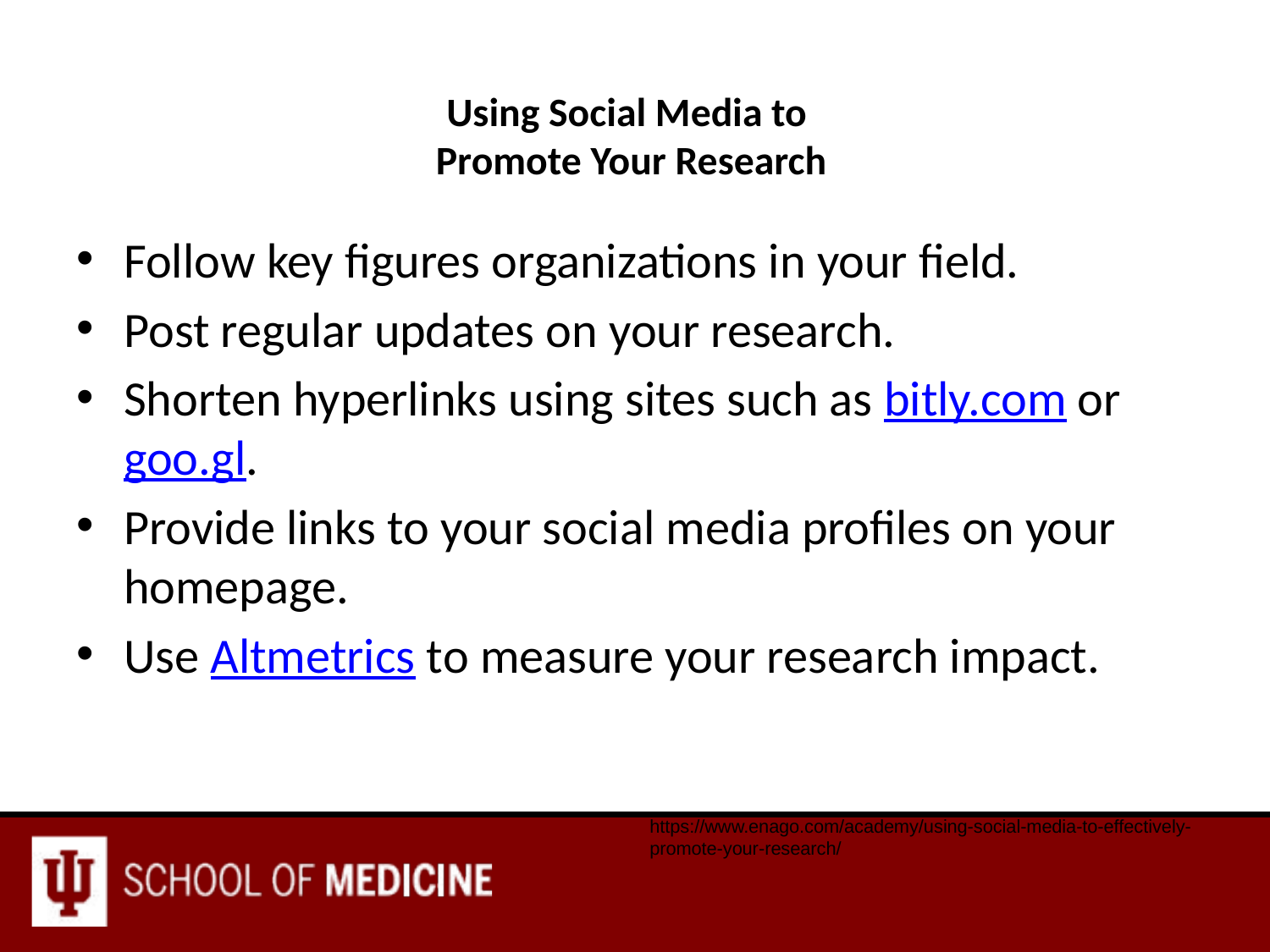

# Using Social Media to Promote Your Research
Follow key figures organizations in your field.
Post regular updates on your research.
Shorten hyperlinks using sites such as bitly.com or goo.gl.
Provide links to your social media profiles on your homepage.
Use Altmetrics to measure your research impact.
https://www.enago.com/academy/using-social-media-to-effectively-promote-your-research/

## Slide 24
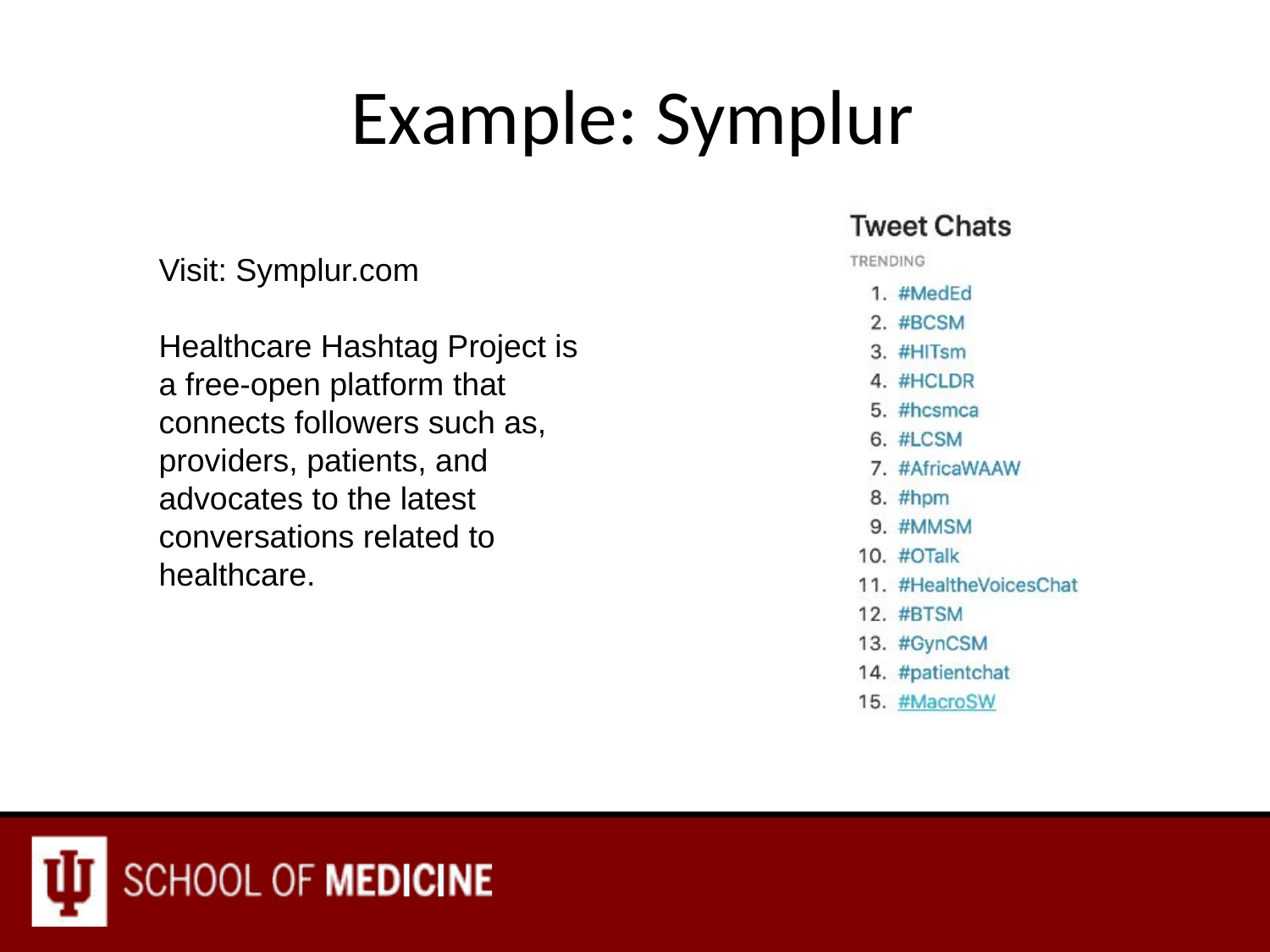

# Example: Symplur
Visit: Symplur.com
Healthcare Hashtag Project is a free-open platform that connects followers such as, providers, patients, and advocates to the latest conversations related to healthcare.

## Slide 25
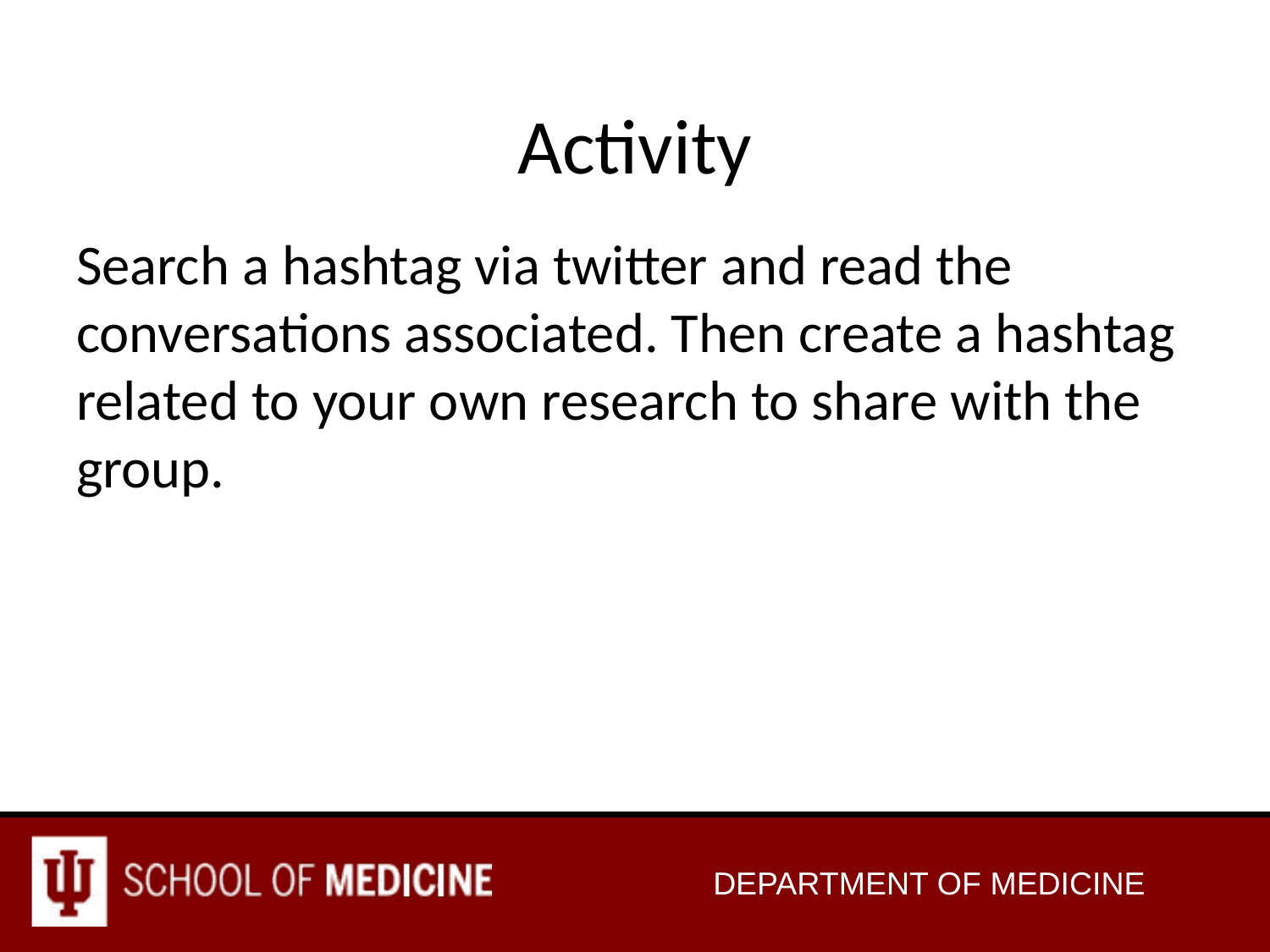

# Activity
Search a hashtag via twitter and read the conversations associated. Then create a hashtag related to your own research to share with the group.
DEPARTMENT OF MEDICINE

## Slide 26
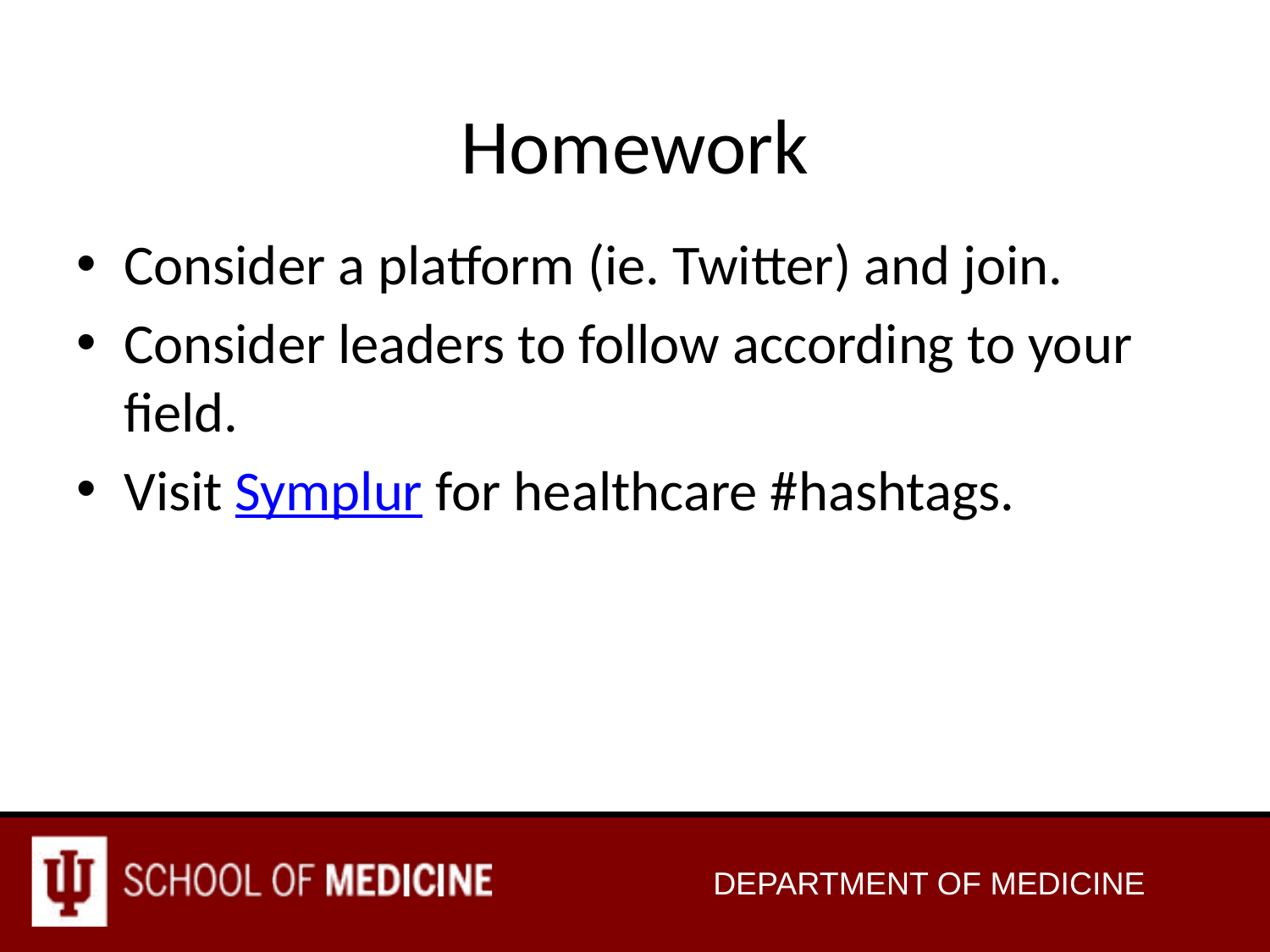

# Homework
Consider a platform (ie. Twitter) and join.
Consider leaders to follow according to your field.
Visit Symplur for healthcare #hashtags.
DEPARTMENT OF MEDICINE

## Slide 27
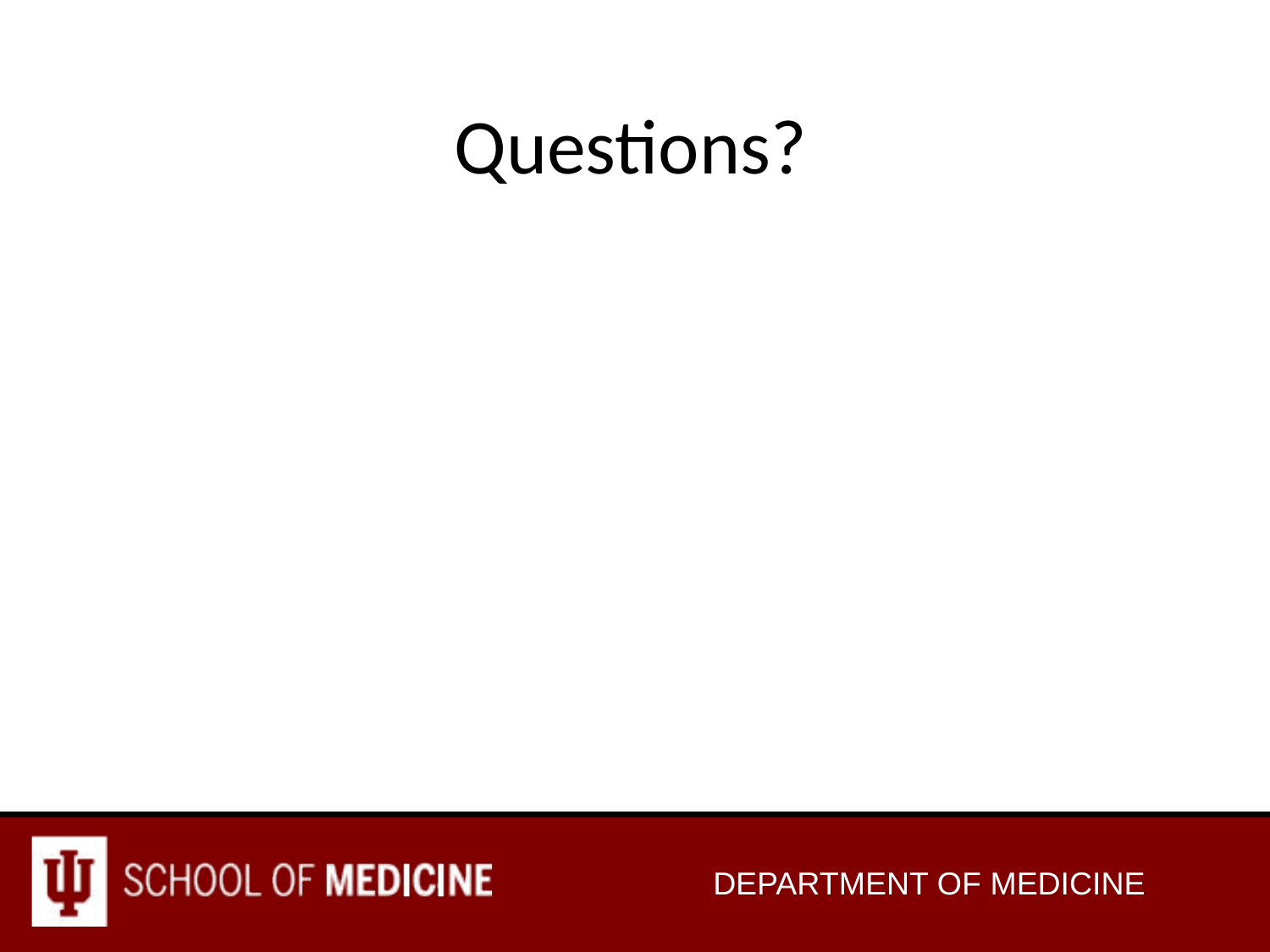

# Questions?
DEPARTMENT OF MEDICINE
