# Supplementary material for: The Power of Social Media in the Promotion and Tenure of Clinician Educators
Source: MedEdPORTAL. 2020 Aug 10;16:10943. doi: 10.15766/mep_2374-8265.10943 (PMC7431188; doi:10.15766/mep_2374-8265.10943)
Supplement: Supplementary file 1 — Content Slides.pptxEvaluation Form.docx [file mep_2374-8265.10943-s001.zip › B. Evaluation Form.docx]

| **Tweets, Likes, & Shares: The Power of Social Media & Promotion** | | | | | | |
| --- | --- | --- | --- | --- | --- | --- |
| *Directions:* We would appreciate your help in improving future workshops by letting us know how you feel about this one. Using the 5-point Likert scale, please ***circle*** your response to each of the following  items: | **Strongly Agree** | **Agree** | **Undecided** | **Disagree** | **Strongly Disagre e** | **N/A** |
| **1. The information presented in this workshop was**  **useful to my professional work.** | 5 | 4 | 3 | 2 | 1 |  |
| **2. As a result of attending this workshop, I am better prepared to turn my current work into**  **scholarship** | 5 | 4 | 3 | 2 | 1 |  |
| **3. My professional work will improve as a result of**  **attending this workshop.** | 5 | 4 | 3 | 2 | 1 |  |
| **4. I would recommend this workshop to a**  **colleague.** | 5 | 4 | 3 | 2 | 1 |  |

1. **Prior to this workshop how familiar were you with the material presented**? (please ***circle*** where you fall on this continuum)

*Previously*

5 4 3 2 1

*Previously*

*very familiar unfamiliar*

1. **How much new information did you learn from this workshop?** (please ***circle*** where you fall on this continuum)

*Learned a Did not learn*

5

4

3

2

1

*great deal of anything new*

*new information*

1. **What will you incorporate from this workshop into your work?**
2. **What part of the workshop resonated for you? How so?**
3. **What would you have liked to hear more about?**
4. **What suggestions do you have to improve this workshop so it would be more beneficial to your professional development?**
