# Supplementary material for: The Power of Social Media in the Promotion and Tenure of Clinician Educators
Source: MedEdPORTAL. 2020 Aug 10;16:10943. doi: 10.15766/mep_2374-8265.10943 (PMC7431188; doi:10.15766/mep_2374-8265.10943)
Supplement: Supplementary file 1 — Content Slides.pptxEvaluation Form.docx [file mep_2374-8265.10943-s001.zip › _Educational_Summary_Report_10943.pdf]

MedEdPORTAL® The AAMC Journal of Teaching and Learning Resources

Original Publication Open Access

The Power of Social Media in the Promotion and Tenure of Clinician Educators

Sylk Sotto-Santiago, EdD, MBA, MPS*, Sacha Sharp, MA, Jacqueline Mac, MA

*Corresponding author: ssotto@iu.edu

Abstract

Introduction: Social networking sites (or social media [SM]) are powerful web-based technologies used to bolster communication. SM have changed not only how information is communicated but also the dissemination and reception of a variety of topics. This workshop highlighted the benefits of SM for clinician educators. The use of SM was explored as a way to maximize opportunities for clinician educators to network, establish themselves as experts, and build a national reputation leading to promotion. The target audience for this submission is faculty developers who would like to implement a similar workshop, and clinician-educator faculty motivated by promotion and advancement. **Methods:** The training workshop involved an interactive session, with approximately 20 minutes of content, 20 minutes of individual and small-group activities, and 15 minutes of large-group discussion. The effectiveness of the workshop was evaluated by asking participants to complete a postsession survey of SM knowledge, attitude, and action. **Results:** Survey responses ($n = 14$) demonstrated an increase in participants' knowledge of SM platforms, ability to identify benefits of SM, skills to disseminate their work, and eagerness to build their personal brand. **Discussion:** This workshop provided a foundation for clinician educators to think strategically about SM use in ways that highlight access to a broader network of colleagues and potential collaborators and that influence the impact of publications and work.

Keywords
Clinician Educators, Social Media, Promotions and Tenure, Professional Brand, Career Choice, Communication Skills, Faculty Affairs, Faculty Development, Minority Recruitment, Publishing/Scholarship

Educational Objectives

By the end of this activity, faculty participants will be able to:

1. Critically examine the benefits and challenges associated with social media (SM) use.
2. Determine how they can use various SM platforms to promote scholarship dissemination and building national presence.
3. Engage in activities that allow for marketing themselves using SM.
4. Use SM to promote both the conduct and dissemination of research.

Introduction

Given the various responsibilities and expectations assumed by clinician educators, it is important to explore innovative resources that, when used effectively, can assist in reaching professional goals. Clinician educators in academic medicine devote their time to caring for diverse patient populations in various settings while also teaching and supervising trainees.[1] Although great progress has occurred since the recognition of clinician-educator faculty tracks, challenges prevail in encouraging and supporting their promotion and tenure.[2-7] Generally, clinician-educator faculty are evaluated on the basis of teaching competence, published and retrievable scholarship, and local, regional, and national reputation. However, clinician educators face barriers to scholarship production, including lack of time, insufficient skills, and limited access to mentoring.[7,8] Specifically, clinician-educator faculty struggle to find time to write and develop their scholarship and to maximize venues for establishing their expertise and presence at regional and national levels.

A promising way for clinician educators to enhance the exposure of their scholarship and elevate visibility at the regional and national levels is through the use of social media (SM). SM have changed not only how information is communicated but also the dissemination and reception of a variety of topics.[9] SM are

Citation:
Sotto-Santiago S, Sharp S, Mac J. The power of social media in the promotion and tenure of clinician educators. *MedEdPORTAL*. 2020;16:10943. https://doi.org/10.15766/mep_2374-8265.10943

Copyright © 2020 Sotto-Santiago et al. This is an open-access publication distributed under the terms of the Creative Commons Attribution license.

powerful web-based technologies with high data-distribution capabilities that allow for the immediate transfer of data, while also maintaining robust metrics concerning personal connections and reach.[10] There exists a rich landscape of multidimensional SM platforms with different functionalities to meet the needs of diverse users.[11] A majority of Americans retrieve their news and entertainment from SM and use SM platforms to learn about global and societal events.[12]

In 2017, a pivotal article by Freitag, Arnold, Gardner, and Arnold highlighted the benefits of SM with regard to academic medicine.[13] For example, Freitag et al. found SM to directly influence the impact of publications by garnering a larger reach. Other scholars have reemphasized the advancement of scholarship, broadness of the audience, and possibilities to enact change.[14,15] For example, in 2017, the AAMC highlighted that it had 20 active Twitter handles totaling over 146,000 followers, six Facebook pages, and accounts on platforms including YouTube, Instagram, Tumblr, LinkedIn, and WordPress. These SM venues reach members, constituents, policy makers, medical school applicants and students, media, and the public.[16,17] Through that reach, researchers could conduct information exchanges and connect with other prominent researchers in their areas of interest. SM encourage collaborative involvement, early development, and rapid dissemination of project ideas, along with data collection and validity. Alternatively, the exposure SM provide allows researchers to generate calls to action around particular movements. The information exchanges available through SM also serve as a critical link for those in underserved areas who would otherwise not have access to the latest information from journals, conferences, courses, and textbooks. Moreover, SM offer a rich network of connections with other pathologists, physicians, and patients. These connections can prove invaluable for personal and career advancement, education, and research.

Concerning implications for promotion and tenure, SM and their influence have been increasingly recognized by academic institutions. Academic institutions themselves are quite active on SM, which assist organizations in branding and also in reaching important constituents and recruiting efforts. Similarly, SM offer individuals a unique opportunity to brand themselves within specialized niches while highlighting experience and expertise. Additionally, SM presentations at national meetings represent a growing and ever-popular form of education. According to Cabrera, Roy, and Chisolm, SM-based scholarship and SM portfolios are now being recognized by institutions of academic medicine as relevant and impactful contributions to promotion and tenure packages.[10] Institutions have recognized the limitations of traditional journals related to reach and impact and therefore have looked to SM to fill in the gaps. Moreover, academic institutions are beginning to establish policies and guidelines to account for SM productivity.

Given the influence of SM and its implications for promotion and tenure, training programs and research that encourage SM use are pertinent. While the field of academic medicine often assumes there is adequate understanding around the capabilities of SM (i.e., the use of Twitter handles and hashtags incorporated in research presentations and educational materials), only a small proportion of professionals are using SM to brand themselves and promote their work. For example, a search on *MedEdPORTAL* using the keywords *social media* and *Twitter* yielded 29 results, of which only one focused on the use of SM and the potential impact in patient-physician relationships, interpersonal relationships at work, institutions' reputations, and the public's trust in health care professionals.[18] However, there is a dearth of research concerning SM use in the promotion and tenure process, related to building and maintaining a personal brand, leveraging specific SM tools, and strategies for advancing research through SM modalities.

This workshop addressed the gap both in the extant literature as well as in the education of faculty on the importance of SM and their potential uses. The workshop went beyond using SM as relationship-building tools and emphasized SM as currency that could enhance promotion and tenure portfolios. Particularly for underrepresented or historically marginalized faculty in medicine, SM create a space for individuals to come together and express perspectives that often go unacknowledged in predominately White spaces.[6] Hence, this session encouraged the use of SM and trained faculty in the ways of maximizing opportunities to network, establishing themselves as experts, and building a national reputation leading to promotion and tenure. The target audience was faculty developers who wanted to implement a similar workshop as well as clinician-educator faculty.

Methods

Participants for this workshop ranged from clinician educators interested in initiating SM accounts to those who already had SM accounts and were interested in leveraging those spaces for promotion. The participants were recruited using marketing materials that were sent directly to members of the department of medicine, as well as individuals outside the department.

Participants were encouraged to have at least one SM account prior to participation in the workshops. Twitter was suggested for

Copyright © 2020 Sotto-Santiago et al. This is an open-access publication distributed under the terms of the Creative Commons Attribution license.

the reasons described in the Introduction. However, as part of this content the facilitators discussed several popular platforms, such as Facebook, LinkedIn, Instagram, and YouTube, among others. Participants were provided time to sign up/in to their SM accounts.

According to Indiana University, SM can be a "tricky area to navigate because of privacy, free speech and copyright issues."[19] University policy prohibits those affiliated with the institution from "post[ing] any intentionally malicious, defamatory, degrading or hateful material."[19]

This session strictly differentiated between personal and professional use, emphasizing a positive professional presence. Faculty developers should address their own institution's SM policies.

Facilitators should be familiar with their own institution's promotion and tenure criteria. Facilitators may also find it beneficial to partner with a representative from their office of faculty or academic affairs to address specific questions related to promotion and tenure. This step offered an opportunity to provide professional development and reinforce support and motivation for promotion (Appendix A).

Small-Group Activity

In pairs, participants discussed which SM platforms they regularly used. It was important to once again discuss the difference between personal and professional use. Appendix A, slides 13-18, specifically discussed SM platforms. Facilitators may focus on platforms they are most comfortable discussing, providing an overview of their own experiences. Selecting a platform may be an individual act and should provide an opportunity to connect with other participants.

Appendix A, slides 19-24, focused on Twitter use for connecting with collaborators, colleagues, and experts. In addition, it discussed potential uses for research purposes.

Evaluation

Upon conclusion of the workshop, participants were asked to complete the evaluation (Appendix B). This evaluation survey included 10 items. The instrument evaluated overall workshop content, as well as offering the opportunity to provide feedback on information presented and postworkshop attitudes toward SM utilization. The instrument mirrored professional development and diversity evaluation forms developed by our office of faculty affair's program evaluation and assessment team and aligned with other workshop evaluations developed and utilized in the department of medicine. Completion of the anonymous survey was estimated at 5 minutes and collected upon completion. Descriptive statistics were generated to evaluate percent agreements.

Qualitative responses were analyzed through open and thematic coding. One facilitator identified themes in these responses and discussed them with members of the team. Facilitators came together to achieve consensus and generate collective themes. The combination of survey findings and themes generated form the basis of our Discussion.

The appendices include our PowerPoint slides (Appendix A) and evaluation form (Appendix B). Slides in this curriculum contain scripts and links to relevant information. The materials required include a smartphone or laptop with internet access.

The optimal length of this workshop was 90 minutes. However, it could be tailored to 60 minutes based on resources. A suggested time line for the workshop from Appendix A is as follows:

- Slides 1-4: 15 minutes (introduction and large-group activity).
- Slides 5-18: 20 minutes.
- Slide 19: 7 minutes (small-group activity).
- Slides 20-24: 15 minutes.
- Slide 25: 7 minutes (activity).
- Slide 26: 7 minutes (activity).
- Slide 27: 10 minutes (answer questions and encourage after-workshop homework or include in workshop dependent on time allotted).
- Postworkshop evaluation: 9 minutes.

Results

This workshop was provided twice and implemented in the department of medicine. It was facilitated by the authors; the vice chair of the department of medicine focused on faculty affairs, professional development, and diversity, with colleagues focusing on career mentoring. Participants included early-career faculty, postdoctoral students, and professional staff who supported faculty with administrative duties. A total of 25 participants signed up for the workshop. A total of 14 completed evaluations were reviewed. The overall workshop evaluation was positive. Additionally, qualitative data suggested that the workshop was well received and helpful.

Survey responses indicated that 21% of participants strongly agreed, with another 57% agreeing, that the information presented was useful to their professional work. As a result of

Copyright © 2020 Sotto-Santiago et al. This is an open-access publication distributed under the terms of the Creative Commons Attribution license.

Table. Workshop Evaluation ($n = 14$)

Item	No. (%)				
	Strongly Agree	Agree	Undecided	Disagree	Strongly Disagree
The information presented in this workshop was useful to my professional work.	3 (21%)	8 (57%)	3 (21%)	0 (0%)	0 (0%)
As a result of attending this workshop, I am better prepared to turn my current work into scholarship.	2 (14%)	6 (42%)	2 (14%)	0 (0%)	0 (0%)
My professional work will improve as a result of attending this workshop.	3 (21%)	7 (50%)	4 (28%)	0 (0%)	0 (0%)
I would recommend this workshop to a colleague.	6 (42%)	7 (50%)	3 (21%)	0 (0%)	0 (0%)

attending the workshop, participants felt that they could take their current work into scholarship, with 14% strongly agreeing and 42% agreeing. A collective 92% would recommend this workshop to colleagues (42% strongly agreed, 50% agreed; see the Table). In addition, the majority of participants seemed to be somewhat familiar with the material presented (43%) based on a 5-point Likert scale (1 = *previously unfamiliar,* 5 = *previously very familiar*; see Figure 1). The majority of participants also reported learning a great deal of information, based on a 5-point Likert scale (1 = *did not learn anything new,* 5 = *learned a great deal of new information*; see Figure 2).

Qualitative data responses indicated considerable endorsement for the use of SM, including incorporating lessons learned. Some comments included: "Will try to disseminate my research on Twitter," "Not to be dormant on social media platforms," and "Need to post more regularly." Participants were asked, "What part of the workshop resonated for you?" They answered, "Engaging more in social media" and "Looking for hashtags and LinkedIn blog publishing feature." For another individual, making the distinction between personal and professional accounts was important. Other participants expressed interest in learning more about how to create their brand and learned more about the benefits of growing this exposure.

Discussion

SM platforms are powerful web-based technologies that have changed how people communicate. Furthermore, the omnipresence of SM and their existence on smartphones have allowed for communication unrestricted by proximity and led to profound sociocultural shifts.[20] This communication shift can be directed towards the maximization of opportunities for clinician educators to network, establish themselves as experts, and build a national reputation, among others. All of these aspects are important to promotion and tenure. The purpose of this workshop is to provide a foundation by which to think strategically about SM use in ways that highlight benefits, such as access to a broader network of colleagues and potential collaborators, influencing the impact of publications, and reaching individuals who otherwise would not have access to critical information. In addition, these aspects all translate into key aspects of promotion and tenure evaluations, such as demonstrating a national or international presence, and the potential collaborations leading to

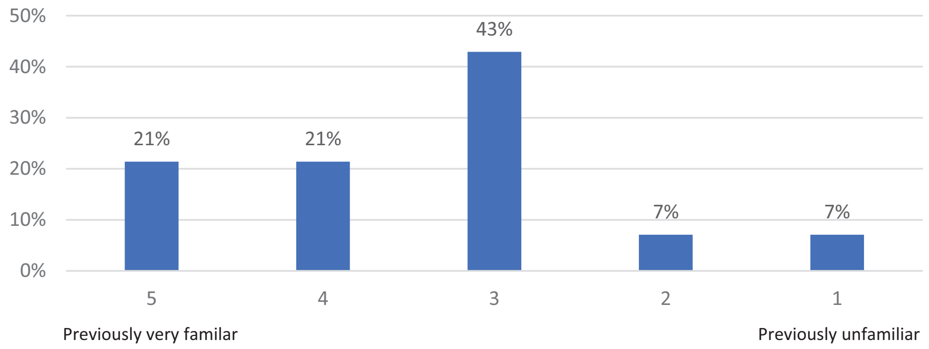

Figure 1. Familiarity with material prior to the workshop.

Copyright © 2020 Sotto-Santiago et al. This is an open-access publication distributed under the terms of the Creative Commons Attribution license.

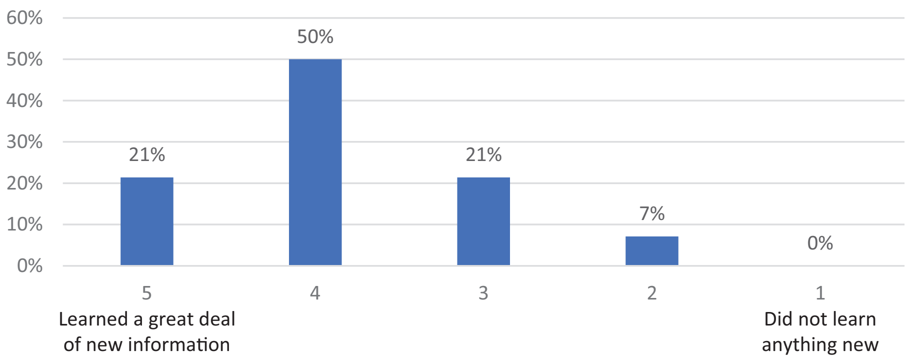

Figure 2. Information acquisition as a result of the workshop.

research projects and publications, scholarships, and curriculum development. Participation in conversations and SM networking can lead to invitations for talks, grand rounds, and submissions to conferences or journals, including as reviewers.

Activity participation, responses, and feedback provided reassurance that alternative ways to disseminate work and magnify voices are at the core of the reasons why this work is done: to advance clinical care, educate the next generation of physicians and scientists, and promote research innovation. Evaluation results suggested not only a better understanding of SM but also a desire to be more specific on strategies available based on areas of interest that individuals would like to promote. For example, based on participation and qualitative comments collected, participants wanted to learn more about other platforms and be more specific about their personal brand. These are important aspects, and we are encouraged to provide this targeted content. This workshop utilizes learner's knowledge of popular networks but shifts the lens so individuals can evaluate their own professional activity and become intentional about what they post in SM.

There were a few lessons learned that we would address in future sessions, and we encourage facilitators to consider these as recommendations. First, one of the comments included a request for more "staff content." This leads us to believe that clinician educators who have administrative assistant support would like also for their staff to be familiar with these strategies and help in building their own brand, not just the department's. Second, depending on audience familiarity with SM platforms, facilitators may choose to also focus on particular ones, such as LinkedIn, since it has blogging features, or academic social networks (e.g., Academia, Research Gate). Lastly, given the blurred line of personal versus public in postings by faculty in academia, it would be wise to reemphasize what is appropriate professional behavior and how to protect oneself from unfortunate consequences of perceived controversial posts.

Implementation challenges included evaluating the level of comfort with some of the SM tools discussed. The range of comfort and current utilization of SM modalities can vary considerably amongst participants. Moreover, the line between personal and professional use can be blurred and challenging for them. It is important for participants to commit to the SM application that they choose and create boundaries that make sense based on their individual goals.

Limitations

Twenty-five individuals participated in this workshop, and survey responses represented close to 60% of them. Selection bias may have been introduced based on the voluntary participation of individuals who were already interested in this topic. Given that those most interested were in early-career faculty ranks, the workshop might be more relevant to those developing a professional identity and professional brand. Mid-career to senior faculty may be interested in other aspects of SM, such as research opportunities. We look forward to expanding this workshop to those in other faculty tracks and areas of excellence. Moreover, SM have created a vast network that can quickly spread information and mobilize individuals for health advocacy efforts.[21-24] The power of SM in this context was acknowledged, and facilitators were also encouraged to discuss the benefits of advocacy. In addition, although the workshop is targeted to clinical faculty, there can be close alignment with faculty in all tracks. Lastly, this workshop would benefit from a more definite assessment of SM use and knowledge prior to content delivery. Future workshops will include an instrument assessing this, instead including such items in the postsurvey.

Copyright © 2020 Sotto-Santiago et al. This is an open-access publication distributed under the terms of the Creative Commons Attribution license.

Future Directions
SM platforms offer an opportunity to advance medical education by sharing with a community of colleagues and experts. However, future workshops should also focus on the challenges of inappropriate activity or activity that may challenge perceptions of professionalism while giving participants tools to address negative attention.

Appendices

A. Content Slides.pptx

B. Evaluation Form.docx

All appendices are peer reviewed as integral parts of the Original Publication.

Sylk Sotto-Santiago, EdD, MBA, MPS: Assistant Professor of Medicine, Department of Medicine, Indiana University School of Medicine

Sacha Sharp, MA: Associate Director of Career Development, Medical Student Affairs, Indiana University School of Medicine

Jacqueline Mac, MA: Graduate Director of Faculty Affairs and Diversity, Department of Medicine, Indiana University School of Medicine; ORCID: http://orcid.org/0000-0002-4073-6819

Disclosures
None to report.

Funding/Support
None to report.

Ethical Approval
Reported as not applicable.

References

1. Shepard D. Increasing awareness—the clinician-educator track in academic medicine. *Hematologist*. 2010;7(3):13.
2. Geraci SA, Thigpen SC. Tenure and the faculty physician. *Am J Med Sci*. 2017;353(2):145-150. https://doi.org/10.1016/j.amjms.2016.12.008
3. Levinson W, Rubenstein A. Integrating clinician-educators into academic medical centers: challenges and potential solutions. *Acad Med*. 2000;75(9):906-912. https://doi.org/10.1097/00001888-200009000-00012
4. Glick TH. How best to evaluate clinician–educators and teachers for promotion? *Acad Med*. 2002;77(5):392-397. https://doi.org/10.1097/00001888-200205000-00008
5. Kumar K, Roberts C, Thistlethwaite J. Entering and navigating academic medicine: academic clinician-educators' experiences. *Med Educ*. 2011;45(5):497-503. https://doi.org/10.1111/j.1365-2923.2010.03887.x
6. Geraci M, Buckley J, Sotto S. Career development for clinician educators securing promotion. Webinar presented at: Alliance for Academic Internal Medicine, Clerkship Directors on Internal Medicine; September 28, 2016; Indianapolis, IN.
7. Chapman T, Carrico C, Vagal AS, Paladin AM. Promotion as a clinician educator in academic radiology departments: guidelines at three major institutions. *Acad Radiol*. 2012;19(1):119-124. https://doi.org/10.1016/j.acra.2011.09.013
8. Reader S, Fornari A, Simon S, Townsend J. Promoting faculty scholarship—an evaluation of a program for busy clinician-educators. *Can Med Educ J*. 2015;6(1):e43-e60. https://doi.org/10.36834/cmej.36666
9. Sharp S. Applying critical theories to social media mining and analysis: #WokeAcademy. In: Kennedy E, Qian Y, eds. *Advancing Educational Research With Emerging Technology*. IGI Global; 2020:78-98.
10. Cabrera D, Roy D, Chisolm MS. Social media scholarship and alternative metrics for academic promotion and tenure. *J Am Coll Radiol*. 2018;15(1):135-141. https://doi.org/10.1016/j.jacr.2017.09.012
11. Bik HM, Goldstein MC. An introduction to social media for scientists. *PLoS Biol*. 2013;11(4): e1001535. https://doi.org/10.1371/journal.pbio.1001535
12. Greenwood S, Perrin A, Duggan M. Social Media Update 2016. Pew Research Center. November 11, 2016. Accessed June 22, 2020. https://www.pewresearch.org/internet/2016/11/11/social-media-update-2016/
13. Freitag CE, Arnold MA, Gardner JM, Arnold CA. If you are not on social media, here's what you're missing! #DoTheThing. *Arch Pathol Lab Med*. 2017;141(11):1567-1576. https://doi.org/10.5858/arpa.2016-0612-SA
14. Gallo T. Congratulations! Your article has been accepted. Now what? Media, social media, and other outlets for promoting your work. *Acad Med*. 2016;91(12):e9. https://doi.org/10.1097/ACM.0000000000001395
15. Sterling M, Leung P, Wright D, Bishop TF. The use of social media in graduate medical education: a systematic review. *Acad Med*. 2017;92(7):1043-1056. https://doi.org/10.1097/ACM.0000000000001617
16. Gallo T. Twitter is trending in academic medicine. Association of American Medical Colleges. February 20, 2017. Accessed March 12, 2019. https://www.aamc.org/news-insights/twitter-trending-academic-medicine
17. Bruns A, Burgess JE. The use of Twitter hashtags in the formation of ad hoc publics. In: Bruns A, De Wilde P, eds. *Proceedings of the 6th European Consortium for Political Research (ECPR)*

Copyright © 2020 Sotto-Santiago et al. This is an open-access publication distributed under the terms of the Creative Commons Attribution license.

General Conference. European Consortium for Political Research; 2011.

18. Robertson M, Shoss MK, Broom MA. Social media: social intelligence training module. *MedEdPORTAL*. 2016;12:10442. https://doi.org/10.15766/mep_2374-8265.10442

19. Social media: legal information, policies & incidents. Indiana University. Accessed May 2, 2019. https://brand.iu.edu/tools/social-media/policies-legal.html

20. Healthcare Hashtag Project. Symplur. 2019. Accessed May 2, 2019. https://www.symplur.com/healthcare-hashtags/

21. Brock A. “Who do you think you are?”: race, representation, and cultural rhetorics in online spaces. *Poroi*. 2009;6(1):15-35. https://doi.org/10.13008/2151-2957.1013

22. Junco R, Elavsky CM, Heiberger G. Putting Twitter to the test: assessing outcomes for student collaboration, engagement and success. *Br J Educ Technol*. 2013;44(2):273-287. https://doi.org/10.1111/j.1467-8535.2012.01284.x

23. Manago AM. Identity development in the digital age: the case of social networking sites. In: McLean KC, Syed M, eds. *The Oxford Handbook of Identity Development*. Oxford University Press; 2014:508-524.

24. Ventola CL. Social media and health care professionals: benefits, risks, and best practices. *P T*. 2014;39(7):491-499, 520.

Received: May 20, 2019
Accepted: January 25, 2020
Published: August 10, 2020

Copyright © 2020 Sotto-Santiago et al. This is an open-access publication distributed under the terms of the Creative Commons Attribution license.
